# Secretory molecules from secretion systems fine-tune the host-beneficial bacteria (PGPRs) interaction

**DOI:** 10.3389/fmicb.2024.1355750

**Published:** 2024-02-26

**Authors:** Garima Gupta, Puneet Singh Chauhan, Prabhat Nath Jha, Rakesh Kumar Verma, Sachidanand Singh, Virendra Kumar Yadav, Dipak Kumar Sahoo, Ashish Patel

**Affiliations:** ^1^Institute of Biosciences and Technology, Shri Ramswaroop Memorial University, Barabanki, Uttar Pradesh, India; ^2^Microbial Technologies Group, CSIR-National Botanical Research Institute, Lucknow, Uttar Pradesh, India; ^3^Department of Biological Sciences, Birla Institute of Technology and Science, Pilani, Rajasthan, India; ^4^Department of Biosciences, SLAS Mody University of Science and Technology, Sikar, Rajasthan, India; ^5^Department of Biotechnology, School of Energy Technology, Pandit Deendayal Energy University, Gandhinagar, Gujarat, India; ^6^Department of Lifesciences, Hemchandracharya North Gujarat University, Patan, Gujarat, India; ^7^Department of Veterinary Clinical Sciences, College of Veterinary Medicine, Iowa State University, Ames, IA, United States

**Keywords:** beneficial bacteria, PGPR, plant immunity, rhizobia, rhizosphere

## Abstract

Numerous bacterial species associate with plants through commensal, mutualistic, or parasitic association, affecting host physiology and health. The mechanism for such association is intricate and involves the secretion of multiple biochemical substances through dedicated protein systems called secretion systems SS. Eleven SS pathways deliver protein factors and enzymes in their immediate environment or host cells, as well as in competing microbial cells in a contact-dependent or independent fashion. These SS are instrumental in competition, initiation of infection, colonization, and establishment of association (positive or negative) with host organisms. The role of SS in infection and pathogenesis has been demonstrated for several phytopathogens, including *Agrobacterium, Xanthomonas, Ralstonia,* and *Pseudomonas*. Since there is overlap in mechanisms of establishing association with host plants, several studies have investigated the role of SSs in the interaction of plant and beneficial bacteria, including symbiotic rhizobia and plant growth bacteria (PGPB). Therefore, the present review updates the role of different SSs required for the colonization of beneficial bacteria such as rhizobia, *Burkholderia, Pseudomonas, Herbaspirillum*, etc., on or inside plants, which can lead to a long-term association. Most SS like T3SS, T4SS, T5SS, and T6SS are required for the antagonistic activity needed to prevent competing microbes, including phytopathogens, ameliorate biotic stress in plants, and produce substances for successful colonization. Others are required for chemotaxis, adherence, niche formation, and suppression of immune response to establish mutualistic association with host plants.

## 1 Introduction

Plants interact with a myriad of microbes, commencing pathogenic, mutualistic, or commensal relationships. This partnership may depend on various external factors (e.g., environment), intrinsic factors, and the signal exchange between the two (Tseng et al., 2009). Although a thin demarcation exists between symbiont and pathogen, a pathogenic association can become symbiotic and *vice-versa*. Despite the common features shared by various groups, signal molecules and resulting networks might be involved in different kinds of associations. Various findings indicate the role of protein secretion in gaining a clear-cut understanding of plant-microbe interactions, as they play a foremost role in fine-tuning the interaction between the two partners (Tampakaki, 2014). The secreted proteins are delivered in the immediate environment or host environment through one or more dedicated systems, called secretion systems (SS). Evolutionary studies of SS infer that both pathogenic and beneficial bacteria have co-evolved to successfully interact with the host plant.

The secretion system is the gateway for the disbursement of proteins from the bacterial cytoplasm into different environments and neighboring cells (prokaryotic or eukaryotic). It is also required for manipulating the host for establishment inside the host cells (Maffei et al., 2017). SS plays a role in a multitude of processes, such as the intoxication of host cells, nutrient acquisition, evasion of host immune mechanisms, pathogenesis, and interbacterial competition (McQuade and Stock, 2018). Almost all bacteria have different SS responsible for secreting a wide array of substrates, and some bacterial species can secrete only one or a few proteins. There are several categories of SS in bacteria based on their architecture and modes of action. Depending on the type of secretion system, they can transport protein substrates across a single-, double-, or even three-layered phospholipid membranes (reviewed in Green and Mecsas, 2016; Klein et al., 2020).

There is a wide array of SS ranging from Type I to Type XI. These systems may supply proteins from cytoplasm straight away into the target cells (T3SS, T4SS, and T6SS), or toward the extracellular space (T1SS, T2SS, two partner systems, and autotransporters) or into the periplasm (the Sec and Tat systems) (McQuade and Stock, 2018). Bacterial effectors traverse membranes in two steps: First, via Sec or Tat SS passed on to periplasm, and in the next step, they are fetched across the outer membrane via other transport systems (T2SS, T5SS, and T9SS). This two-step procedure is termed Sec- or Tat-dependent secretion. Numerous proteins are secreted through “Sec- or Tat”-independent protein secretion channels by traversing both the inner and outer bacterial membranes. So, SS, such as T1SS, T3SS, T4SS, and T6SSs, conduct a one-step process leading to the delivery of effectors into the medium (T1SS) or host cells (T3SS, T4SS, and T6SS) (reviewed in Chang et al., 2014; Galán et al., 2014; Walker et al., 2017; Sana et al., 2020).

Many SS have common features, and some are unique, which might be due to evolutionary processes, including multiple events of gene expunction, gene accrual, and horizontal gene transfer (HGT), adding new features to the secretory machines from the previous one. These co-option procedures depend on the complexity of SS, distinguished features from ancestral machines, availability of genetic material to tinker, and mechanisms of effector recognition or discrimination. It further leads to the discovery of new SS and distinguishes additional features in the recently identified systems (Denise et al., 2020). In a recent study, the experimental knowledge of model protein SS was used to create computational models (using MacSyFinder25) to better understand composition, genetic organization, and identification of SS in bacteria (Abby et al., 2016). A recent study used super-resolution microscopy techniques to understand host–microbe interactions, changes in sub-cellular compartments of live bacteria, architecture, and mechanism of action of SS in bacteria (Gunasinghe et al., 2017; Guo et al., 2022; Li et al., 2023).

The role of different SS in host and plant interaction has been demonstrated in both plant and animal systems and has been reviewed in previous literature (Green and Mecsas, 2016; Qin et al., 2022; He et al., 2023). Researchers reviewed the architecture, transport mechanisms, and roles of specialized SS, mostly in pathogens and their substrates in finding niches, as well as in delivering virulence factors for causing microbial pathogenesis or virulence (Xu and Liu, 2014; Jiang et al., 2023). However, the role of SS in the establishment of beneficial microbe-plant interaction is limited to rhizobia and not thoroughly reviewed. Recently, Lomovatskaya et al. reviewed the recent updates on the organization and operational aspects of SS of plant pathogens and a few beneficial bacteria and their importance in suppressing plant defense responses (Parizad et al., 2016; Lomovatskaya and Romanenko, 2020; Lu et al., 2022). Therefore, the present review updates the mechanistic aspects of the secretion system in chemotaxis, initiation of infection, colonization, and establishment of beneficial bacteria in the host. These beneficial bacteria have huge relevance for enhanced and sustainable agriculture and include symbiotic bacteria such as rhizobia and other plant growth-promoting bacteria (PGPB). Based on the colonization area, these PGPBs can be rhizospheric (PGPR), rhizoplanic, and endophytic. They promote plant growth by directly providing nutrients and phytohormones and indirectly by ameliorating biotic and abiotic stress (Gupta et al., 2012).

## 2 Secretion system in PGPRs: an overview

SS has been reported in a wide spectrum of PGPR (plant growth-promoting bacteria) in the last few decades, which is the main focus of this review paper (Supplementary Table S1). Mostly, Type I and Type II SS are observed in bacterial endophytes (on genome comparison of 11 well-known endophytes) (Reinhold-Hurek and Hurek, 2011), whereas Type III and Type IV SS are more prevalent in pathogenic bacteria (Downie, 2010). Type V SS, an autotransporter, and T6SS systems are very common in endophytes and have several important roles during plant-microbe interactions. Several studies divulged the existence and importance of a secretion system in Rhizobial strains, secreting proteins into the periplasm via leakage in the extracellular growth medium, GEP, TAT system, etc. (Tsyganova et al., 2021). Rhizobia is a group that includes various genera like *Rhizobium*, *Sinorhizobium* (*Ensifer*), *Mesorhizobium*, and *Bradyrhizobium*. Type I, Type III, Type IV, and Type VI SS in a variety of rhizobial strains play an immense role in the process of symbiotic interaction with host plants (reviewed in Fauvart and Michiels, 2008; Tsyganova et al., 2021). Therefore, this review paper will present the current state of research on SS present in various plant growth-promoting bacteria and their role in plant-microbe interaction. The following subsections deal with updates regarding different SS involved in beneficial plant-microbe interaction (Table 1).

**Table 1:** Summary of various secretion systems present in plant growth-promoting bacteria (PGPRs).

Secretion system	Genera	Functions reported	References
SEC	Gram positive: *Bacillus,* Gram negative: *Pseudomonas*, *Rhizobium*, *Klebsiella*, *Enterobacter, Hartmannibacter*, *Nitrospirillum*	Colonization, secrete various proteins	Supplementary Tables S1, S2
TAT	Gram positive: *Bacillus,* Gram negative: *Pseudomonas*, *Rhizobium*, *Mesorhizobium*, *Herbaspirillum*, *Klebsiella*, *Enterobacter, Hartmannibacter*, *Nitrospirillum*	Nitrogen fixation, aerobic and anaerobic growth of bacteria, symbiosis, root nodule development, cell component synthesis, nodulation initiation, secrete symbiosis specific proteins, fimbriae biogenesis and biofilm formation etc	Supplementary Tables S1, S3
T1SS	Gram positive: *Bacillus,* Gram negative: *Pseudomonas*, *Azoarcus*, *Rhizobium*, *Sinorhizobium, Bradyrhizobium, Neorhizobium*, *Kosakonia*, *Xanthomonas*, *Klebsiella*, *Hartmannibacter*, *Burkholderia*	Symbiosis, attachment, nitrogen fixation, infection threads development or senescing plant cells, bacteriocin/antibiotic production, biofilm formation, resistance toward macrolide antibiotic tylosin, secrete metalloprotease, glycosyl hydrolase, cadherins, calcium-binding proteins, and a nucleoside diphosphate kinase, and infection proteins	Supplementary Tables S1, S4
T2SS	Gram-positive: *Bacillus,* Gram-negative: *Pseudomonas*, *Azoarcus*, *Rhizobium*, *Sinorhizobium, Bradyrhizobium, Herbaspirillum*, *Mesorhizobium*, *Dugesia, Kosakonia*, *Xanthomonas*, *Klebsiella*, *Enterobacter, Hartmannibacter*, *Burkholderia*, *Stenotrophomonas*	Cause infection by suppressing the flg22 response	Supplementary Tables S1, S5
T3SS	Gram negative: *Pseudomonas*, *Rhizobium*, *Sinorhizobium, Bradyrhizobium, Dugesia, Herbaspirillum*, *Kosakonia*, *Paraburkholderia*, *Klebsiella*, *Hartmannibacter*, *Burkholderia*	Symbiotic association, enhancing defense responses flagellum-related genes, PR genes suppression, formation of infection thread, nodule formation, biocontrol activity	Supplementary Tables S1, S6
T4SS	Gram negative: *Pseudomonas*, *Azoarcus*, *Rhizobium*, *Sinorhizobium, Ensifer*, *Sinorhizobium, Bradyrhizobium, Mesorhizobium*, *Neorhizobium*, *Kosakonia*, *Paraburkholderia*, *Hartmannibacter*, *Burkholderia*, *Stenotrophomonas*	Conjugation-transfer of symbiotic plasmids, microbial competition, symbiotic infection, ubiquitinylation of plant proteins for degradation via proteasomes, biocontrol	Supplementary Tables S1, S7
T5SS	Gram-negative: *Pseudomonas*, *Rhizobium*, *Klebsiella*, *Paraburkholderia*, *Stenotrophomonas*	Colonization, twitching, swarming, and swimming motilities, Secrete putative adhesin, a surface-adhesion calcium-binding outer membrane-like protein	Supplementary Tables S1, S8
T6SS	Gram-negative: *Pseudomonas*, *Rhizobium*, *Bradyrhizobium, Herbaspirillum*, *Azospirillum, Kosakonia*, *Paraburkholderia*, *Xanthomonas, Klebsiella*, *Pantoea, Enterobacter, Hartmannibacter*, *Burkholderia*, *Stenotrophomonas*	Nitrogen fixation, antibacterial activity, bacterial motility, adaptation, biocontrol, colonization, production of lipids, carbohydrates, and photosynthetic pigments, attachment, IAA production, competition, fitness or niche adaptation	Supplementary Tables S1, S9
T7SS	Gram-positive: *Bacillus,* Gram-negative: *Pseudomonas*	Niche colonization, changing membrane permeability, iron sequestration	Liu et al. (2023)
T8SS	–	–	–
T9SS	Gram-negative: Flavobacterium	Rhizospheric competence, abundance, motility, elicit defense response, secreting carbohydrate hydrolysing enzymes, and disease resistance	Kolton et al. (2016)

### 2.1 General secretary pathway (GEP)

The most highly conserved secretion system proteins traversing the cytoplasmic membrane of bacteria, archaea, and eukarya are general secretion (Sec) and twin-arginine translocation (Tat) pathways. Many components of these two systems (Sec and TAT) are quite common, but they have different mechanisms of protein transport (Green and Mecsas, 2016).

**Figure 1:**
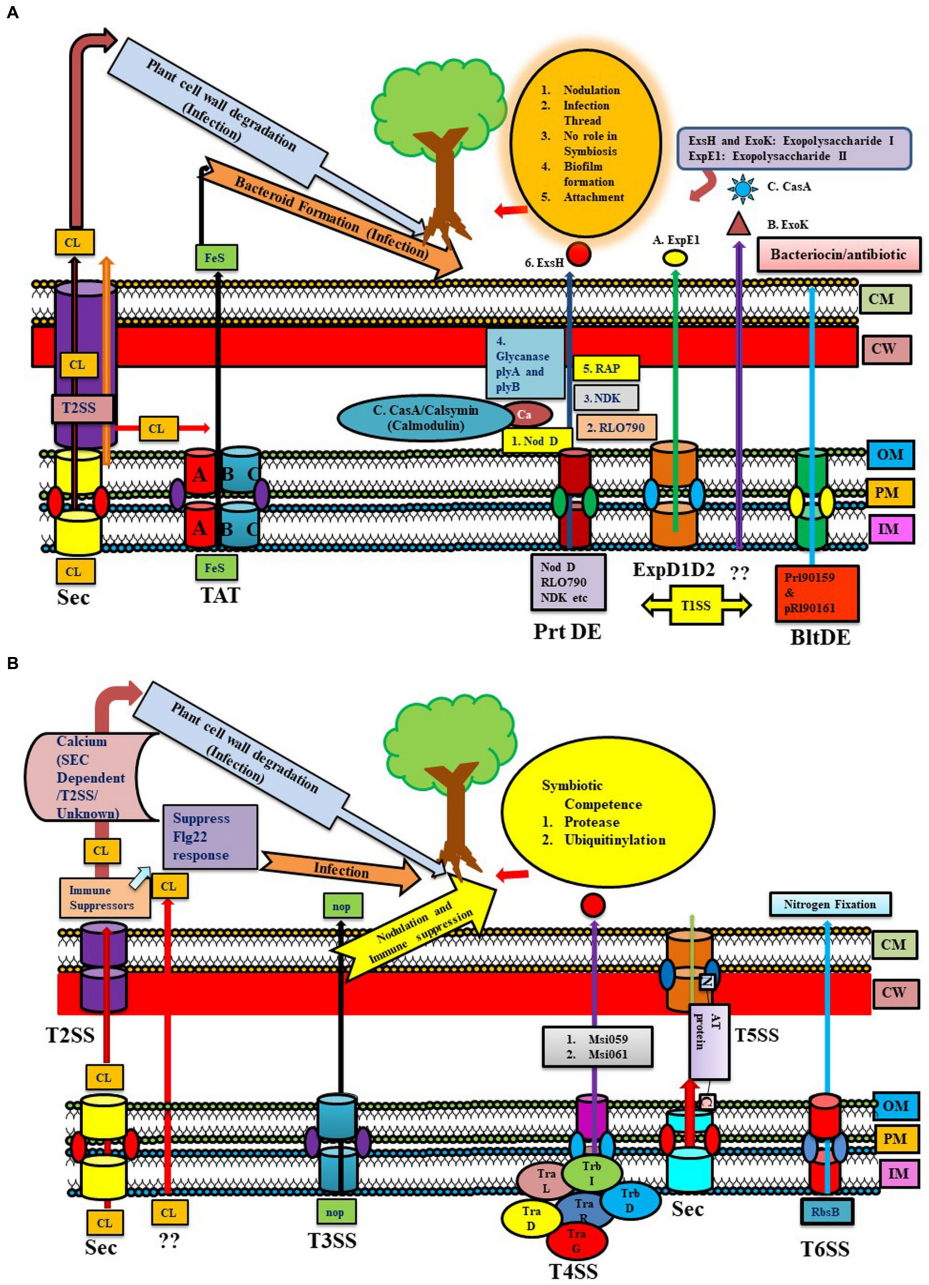
**(A)** Representation of the Sec, TAT, and T1SS (Prt DE, ExpD1D2, BltDE) systems in different Rhizobial species. Sec System: Calcium (CL) export may occur through various pathways like Sec, TAT, Sec via T2SS, or by some unknown mechanism [some part depicted in panel **(B)**] leading to plant cell wall degradation and infection. TAT system (TAT A-BC): exported Iron–sulfur protein helping in infection by forming bacteroids. T1SS have different types of T1SS: 1. Prt DE: transfer NodD, Nucleoside diphosphate kinase (NDK), RLO790 (T1SS), glycanses (plyA and B), RAP, Exs H for exopolysaccharide I, 2. ExpD1D2: transferring ExpE1 for exopolysaccharide II (*Sinorhizobium meliloti*), 3. BltDE T1SS: prl90159 and pRl90161 producing bacteriocin/antibiotic type products. 4. Unknown system: transporting ExoK. **(B)** Representation of T2SS, T3SS, T4SS, T5SS, and T6SS systems in different Rhizobial species. The T2SS system shows calcium export through Sec via T2SS and an unknown pathway. T2SS exports immune suppressors (suppress flg22), which help in infection. T3SS mainly transfers NOP (nodulation outer protein), helping in nodulation and immune suppression (described in Figure 2 elaborately). T4SS system in rhizobial species have similarities to Vir proteins of *Agrobacterium tumefaciens* like *trbD* (*virB3*), *trbI* (*virB10*), and *trbL* (*virB6*). *tra* genes important in conjugative transfer are *traD*, *traR*, and *traG* (*virD4*). *Mesorhizobium loti* strain R7A transfers two proteins via T4SS: Msi059 (protease) and Msi061 (in ubiquitinylation). T5SS system (contains autA, B, C orthologs) secretes autotransporter proteins through the Sec pathway in the periplasm, then via T5SS (c-terminal part is embedded in OM and tries to push the N-terminal part outside the cell). T6SS system exports RbsB involved in nitrogen fixation.

Among PGPR, Rhizobial cellulase enzyme (CelC2) exported via GEP acts on root hair cellulose to cause symbiotic infection in hosts. Mutation of this gene negatively affected its infection capability of *Rhizobium leguminosarum* bv. trifolii in the host plant (Figure 1A). Some researchers have suggested the possibility of an alternative secretion mechanism via T2SS, as some cellulase genes harboring Rhizobia showed the absence of a T2SS system (Robledo et al., 2018; Tsyganova et al., 2021; Supplementary Table S2).

In rhizospheric *Bacillus amyloliquefaciens* FZB42, almost all the extracellular proteins secreted through the Sec system are cleaved by type I signal peptidase and others by lipoprotein-specific signal peptidase II before secretion (Kierul et al., 2015). Researchers distinguished the SS of human pathogenic *P. aeruginosa* and plant symbiont *P. fluorescens* Pf0-1 (Ma et al., 2003). Both of the above pseudomonads showed the presence of outer membrane export systems (MTB (Main terminal branch), FUP (Fimbrial Usher porin), AT (Autotransporter), and TPS (Two partner secretion families)) in several copies (2-9). These two also have three or four ABC-type protein secretory and flagellar SS but lack a type IV system. Unlike *P. aeruginosa, P. fluorescens* showed the absence of a Type III secretion system. Both Pseudomonads have a single copy of each protein of the Sec system. It has been observed that an extra homolog of signal peptidase SPase I (PA1303) is obtained in *P. aeruginosa* but not in *P. fluorescens*.

### 2.2 TAT pathway

Twin arginine translocase (Tat A, B, C) pathway has been located in a range of organisms, from prokaryotes (archaea and bacteria) to eukaryotes (in plant chloroplasts) (Patel et al., 2014; Sana et al., 2020). There are some reports on the TAT system in PGPR, which suggest its role in transferring symbiosis-specific proteins, certain cofactors, and signal peptides and in nitrogen fixation (Meloni et al., 2003). Fourteen species of *Rhizobium* studied pertain to conserved protein clusters like TatA, TatB, and TatC, but not TatE, and each species has a genus-specific Tat system (Supplementary Table S3; Black et al., 2012). Apart from conserved TAT genes in rhizobial species, they also showed significant similarity in intergenic space between *tatA* and *tatB* (31–90 nt) and the arrangement of tat genes and flanking genes, except for an additional ORF in *S. meliloti*.

Recently, in *Mesorhizobium loti* (mlo) MAFF303099, two Rieske Fe/S proteins dependent on the TAT system were reported to induce the nodulation process (Basile et al., 2018). It has a TAT substrate protein (glycosyl hydrolase family) with cellulase activity affecting membrane integrity that might be involved in breaching root hair. Other *M. loti* MAFF303099 Tat substrates include an amidase and two transpeptidases affecting cell wall integrity. Mutation in *tatABC* genes encoding TAT transporter in *R. leguminosarum* leads to loss of nitrogen-fixing ability, possibly due to defective export of iron–sulfur protein in the mutant strains required for bacteroid respiration. It was observed that bacteroids were not formed from the TAT mutant strain, suggesting a role of TAT genes in symbiosis, and also abolished hydrogenase activity at the post-translational level in *Rhizobium* (Meloni et al., 2003). TAT genes are also involved in certain modifications of the cell envelope, synthesis of cell wall components (LPS, EPS, etc), and in aerobic and anaerobic growth of this bacterium, which are important in advanced stages of infection (Meloni et al., 2003). *R. l. bv. Viciae* UPM791 *tatC* mutant form white nodules in peas, cannot fix nitrogen, and lack flagellar proteins (Pickering et al., 2012). Similar to *R. l. bv. viciae* UPM791, the *R. l. bv. viciae* 3841 *tatC* mutant cannot enter the bacteroid region of the infection thread. Unlike *R. l. bv. viciae* UPM791, alive bacteria were recovered from the nodules formed by *R. l. bv. viciae* 3841 *tatC* mutant. The inability of *tatC* mutant to export periplasm electron transport components might be a possible cause of impaired development and nitrogen-fixing capability. On comparing wild type and *tatC* mutant *R. l. bv. viciae* 3841, most TAT substrate proteins are either expressed at a low level or not at all, or increased in leakage of periplasmic contents. The possible reason for membrane leakiness and outer membrane instability is the mislocation of two TAT substrates, AmiA and AmiC (cell wall amidases). Therefore, a wide array of symbiosis-specific proteins is secreted via the TAT pathway (Meloni et al., 2003; Tsyganova et al., 2021). The Tat mutation in various rhizobia strains has been observed to impact multiple cellular processes, including protein secretion, nodule infection, cell division, and activity against antibacterial compounds (Tsyganova et al., 2021; Figure 1A).

In *B. amyloliquefaciens* subsp. Plantarum, the UCMB5113 genome contains 3,693 genes, 298 of which encode secretory pathway proteins. Out of 298 proteins, 149 lacked any transmembrane domain. Another 200 Sec-dependent proteins contained signal peptidase I as a site for cleavage; 87 were detected as lipoprotein consisting SPII/Lsp, and 11 consisted of a TAT motif for protein secretion (Niazi et al., 2014). Comparative studies on human pathogen *P. aeruginosa* and plant symbiont *P. fluorescens* revealed that both have single copies of the *TatA*, *TatB*, and *TatC* genes of the TAT system (Ma et al., 2003). Four in *P. aeruginosa* and two in *P. fluorescens* fimbrial Usher proteins were also observed, which have a role in fimbriae biogenesis and sometimes in biofilm formation.

Based on available literature, it is suggested that the proteins secreted through the TAT pathway are crucial for *Rhizobia-legume* interaction where the secreted proteins are instrumental for infection, nodulation, differentiation of bacterioids, and nitrogen-fixation ability of the symbiont. The presence of genes encoding TAT pathway present in other PGPR indicates its role in microbe-host interaction, which needs to be validated through functional studies.

### 2.3 Type 1 secretion system

Type I or ABC transporter secretion system is the most prominent and well-described secretion system. Similar to rhizobial attachment, infection proteins are transported across the IM and OM through the type I secretion pathway (Alaswad et al., 2019; Supplementary Table S4). The type I system in *Rhizobia* is comprised of three proteins (ArpD/E, HlyB, and PrtDE system) that are observed in one cluster present in all fourteen Rhizobial species. However, in some species, TolC and KEGG Type I secretion proteins orthologs are absent (Black et al., 2012), and their substrates and functions are given in Supplementary Table S4 (Tsyganova et al., 2021). In *R. leguminosarum* bv. *viciae* strain 3,841 (rle), almost four T1SS were found to be encoded by the *toaDE*, *tobDE*, *prsDE*, and *bltDE* genes. For example, NodO, a calcium-binding protein required for infection, is transported by prsDE-encoded secretion system. Generating mutation in *prsD* led to a mutation in NodE due to a shortage in the export of NodO, and further *prsD* mutation reduced the nitrogen-fixation capability of *R. leguminosarum* bv. Viciae (strain A34 but not 3,841) and bv. Trifolii strain TA1. RL0790, a secreted protease exported via T1SS, might be involved in developing infection threads by bacteria or senescing the plant cells. Nucleoside diphosphate kinase (NDK), having no role in symbiosis, is also secreted by T1SS, but their export gets affected in the *prsD* mutant. BltDE secretion system secretes proteins like pRL90159 and pRl90161, producing bacteriocin/antibiotic-type products in the medium (Tsyganova et al., 2021), which might have antagonistic potential against pathogens.

The type I system of *Rhizobium tropici* PRF 81 provides resistance toward macrolide antibiotic tylosin, similar to *Streptomyces fradiae* TlrC protein, and also msbA. Another PRF 81 Type I system export, (1-2)-β-glucanase, exhibiting similarity to NdvA protein of *S. meliloti,* has been identified (López-Baena et al., 2016). This Rhizobium secretes glycanases that can shorten nascent exopolysaccharides of biofilm produced by rhizobia (Marczak et al., 2017). Similar to *Sinorhizobium meliloti, Rhizobium leguminosarum* produces an acidic EPS required for colonization, attachment, and biofilm formation. Two glycanases, PlyA and PlyB, cleave acidic EPS in *R. leguminosarum* and are secreted via PrsD-PrsE type I secretion system. Mutants of *prsD* and *prsE* produce fewer biofilm rings than the wild type, indicating the role of PrsD-PrsE-secreted proteins in biofilm formation in *R. leguminosarum* strain A34. PrsDE T1SS secretes several predicted adhesins (like Rhizobium-adhering proteins ‘Rap’), having an immense role in root adherence and biofilm formation instead of infection (Vozza et al., 2016). Also, rhicadhesin is an important protein factor produced by *R. leguminosarum* for attachment to root hairs, similar to a protein in *Bradyrhizobium* spp. (Tsyganova et al., 2021; Figure 1A). These proteins, along with several other rhizobium-adhering proteins (RAPs)-predicted cadherins (calcium-binding adherence proteins), are secreted via a T1SS across the inner and outer membranes (Vozza et al., 2016). Surprisingly *Rhizobium leguminosarum* Norway has been reported to form inefficient nodules incapable of or showing little nitrogen fixation. Comparing the genome with other Rhizobium strains revealed that the major difference exists in metabolic and protein SS genes rather than *nif* and *fix* genes. It has five putative T1SS systems, where T1SSa, T1SSb, and T1SSc are unique. While T1SSd and T1SSe show almost 90% identity to Rlv 3,841, T1SSa and T1SSc systems are involved in encoding genes related to putative repeats-in-toxin (RTX) toxins. Here, T1SSd proteins showed homology to PrsD and PrsE proteins (like Rlv 3,841) involved in biofilm formation, but T2SS and T3SS are absent, unlike Rlv 3,841 (Liang et al., 2018).

*Sinorhizobium meliloti* forms different kinds of exopolysaccharides, namely succinoglycan (exopolysaccharide I (EPS I)) and galactoglucan (exopolysaccharide II (EPS II)), and either one of them is sufficient for interaction with *Medicago sativa* (Lehman and Long, 2013). Both are responsible for depolymerizing nascent succinoglycan chains. ExoK and ExsH are responsible for exopolysaccharide I production. T1SS secretes ExsH, while ExpE1 is required for EPS II synthesis and secretion via ExpD1D2 T1SS. However, mutational studies revealed that none of these proteins have a significant role in symbiosis (Fauvart and Michiels, 2008).

Through sequence analysis, two T1SS loci were identified in the plant-beneficial *Pseudomonas* strain WCS417 genome, in which the first system encodes lipase and alkaline protease AprA ortholog. Another gene cluster is responsible for synthesizing hemophore HasA ortholog. Similarly, the two other strains of *Pseudomonas* (WCS374 and WCS358) also possess two T1SS gene clusters. The first gene cluster of WCS374 encodes alkaline protease apr. However, little information is available regarding the second gene cluster of WCS374 and the presence of both T1SSs in the WCS358 strain (Berendsen et al., 2015). *Pseudomonas* sp. UW4 possesses many SS, including three fully functional T1SS with their respective putative substrates and one partially functional T1SS containing only MFP and an ABC transporter (Duan et al., 2013). There is a lack of clear understanding of the transfer mechanism in partial T1SS devoid of any OMP (outer membrane protein). In *P. putida* KT2440, LapA protein-synthesizing biofilm is translocated by the type I LapBC secretion system to the cell exterior (Hinsa et al., 2003). *P. putida* W619 has a gene synthesizing an OMP of T1SS (PputW619_5061), a putative adhesin (PputW619_3808), and a surface-adhesion calcium-binding OMP showing less resemblance to LapA (Wu et al., 2011).

Among the nine strains of *Burkholderia phytofirmans* analyzed in a comparative genomic study, eight strains were found to possess T1SS, while six were found to possess T2SS. *B. phytofirmans* PsJN possesses four distinct secretion systems, namely T2SS, T3SS, T4SS, and T6SS. While the presence of only one T1SS gene (membrane fusion protein HlyD) was detected, the inner or outer membrane transport components of T1SS were not found (Mitter et al., 2013). Hence, genomic and functional analysis in Rhizobia and other PGPR, including *B. phytofirmans* and *P. putida*, have demonstrated the undeniable significance of T1SS-secreted proteins in colonization, attachment, infection thread formation, biofilm formation, and antagonism of competing bacteria through production of various proteins including adhesion, cadeherins, glycanases, and EPS-processing enzymes.

### 2.4 Type 2 secretion system

Type II secretion system (T2SS) is mainly present in Gram-negative bacteria, which translocate proteins from the periplasm to the external surroundings through the outer membrane (Douzi et al., 2012; Korotkov et al., 2012; Hay et al., 2017). Very few reports of T2SS are present in plant-beneficial bacteria, which are discussed below. In Rhizobia, cellulase (CelC2) enzyme is an important symbiotic protein required for initial root colonization, which is either secreted by leaking into the growth medium, by general export pathway, or by some other mechanism in case of the absence of a T2SS system (Xie et al., 2012; Figure 1B). T2SS were found only in *Bradyrhizobium*, *M. loti*, and *Sinorhizobium* NGR 234, and were not reported in the other genomes studied (Black et al., 2012). A comparative study between human pathogen *S. maltophilia* K279a and PGPR*. rhizophila* DSM14405T revealed that *S. rhizophila* contains Type II, V, and an incomplete set of Type IV secretion systems. Apart from that, it has a gene stretch containing different genes like *icmF*, *impA*, *Hcp1* family genes, and T6SS *Rhs* genes belonging to Type VI secretion system. Apart from a few similar homologs of Type VI *S. rhizophila* genes, *S. maltophilia* contains genes related to T4SS system playing a role in pathogenesis and horizontal gene transfer (Alavi et al., 2014; Supplementary Table S5).

Root-associated commensals developed host immune suppression mechanisms utilizing non-host specific strategies to gain entry inside the host, unlike pathogens and legume-specific symbionts using T3SS for suppression. Teixeira et al. demonstrated how, with a commensal community of 35 bacterial strains (SynCom35), only *D. japonica* MF79 contained T3SS genes. The *D. japonica* MF79 ΔT2SS mutants could not vanquish the flg22 response in roots of *Arabidopsis*, whereas the ΔT3SS mutant retained the capability to suppress. This indicates the importance of the T2SS system in suppressing the host immune system by commensals to gain entry inside the host (Teixeira et al., 2021).

Berendsen et al. reported the presence of two T2SS loci in *Pseudomonas* WCS417 genome and one locus in WCS374 and WCS358 strains based on BLASTP analysis. WCS358 T2SS locus showed resemblance to Xcp T2SS of *P. aeruginosa* PA01 responsible for secreting PhoX-type phosphatase UxpB under phosphate-limited conditions (Berendsen et al., 2015). In contrast, the activity of other T2SS loci of WCS417 was unknown and identified to be Xcm cluster of *P. putida* GB-1 and KT2440. At the same time, exploring the T2SS of *Pseudomonas* sp. UW4, T2SS genes located within one cluster containing two separate operons were observed (Duan et al., 2013). An endophyte *Enterobacter* sp. SA187, which has all core genes coding for the T2SS, except genes *gspO* (prepilin peptidase) and *gspS* (accessory pilotin), was reported earlier (Andrés-Barrao et al., 2017). Although GspO is required for the function of the secretion system, there are two highly similar genes, including *pilD*, responsible for coding a type IV prepilin-like protein peptidase. So, it seems that *pilD* is used instead of GspO for making a fully functional T2SS secretion system.

In endophytic bacteria, namely *B. phytofirmans-* PsJN colonizing potato plants (Lu et al., 2022), genes for type II and type IV secretion systems were observed (Sheibani-Tezerji et al., 2015). A genome comparison study revealed that, unlike pathogens, PGPB (*Burkholderia* genus) lacked almost all virulence genes like pili production, adhesion, VgrG-5 (T6SS), and toxin biosynthetic gene (Dang et al., 2019). In the case of the Type II secretion system, fewer studies have been done in PGPR so far, and these limited studies indicate the role of T2SS in colonization through secreting hydrolytic enzymes and suppressing host immune responses.

### 2.5 Type 3 secretion system

T3SSs are nano-machines or molecular syringes with a multi-protein complex structure, and their needle length is mandatory for the desired function, although the associated factors of length control are still unknown (Hardoim et al., 2015; Lombardi et al., 2019). Phylogenetic analysis suggests that various TTSSs can be categorized into five groups: (i) the Ysc group, (ii) the Hrp1 group, (iii) the Hrp2 group, (iv) the Inv/Mxi/Spa group, and (v) the Esa/Ssa group. T3SS has been reported mainly in two agronomically important PGPR groups: Rhizobia and Pseudomonads. Rhizobia belongs to the Ysc group and specifically harbors *Rhizobium* Rhc T3SS. Generally, rhizobia contain α-RhcI T3SS and some have an extra T3SS gene cluster of α-RhcII subgroup (unknown function), belonging to Rhizobiales-T3SS family mostly present in pathogenic strains. Previous studies indicate that the differences and commonalities exist between pathogens and beneficial PGPR due to various evolutionary events. For example, certain endophytes lack or have an incomplete T3SS system and can be classified as disarmed pathogens, as they do not elicit hypersensitive responses in the host plant. Unlike mammalian cells, it has been observed that bacteria have long Hrp pilus to penetrate the thick plant cell wall effectively (Hotinger et al., 2021). For example, in Rhizobia (Rhc-T3SS), T2SS/T4P-related secretin gets lost, and only a short rhcC1 aminoterminal remnant gene was found left in T3SS gene cluster, and Tad (Tight Adherence) like secretin (rhcC2) got added into it through recombination events. In *Rhizobium etli*, the *rhcC1* gene is present and either a Tad-like secretin (*rhcC2*) was lost or never acquired (Abby et al., 2016). Similarly, in *Mesorhizobium amorphae* CCNWGS0123, all the required components of T3SS-I were present, but T3SS-II lacked the extracellular elements and both T3SS genes showed opposite effects on exposure to flavonoids (T3SS-I genes upregulated and T3SS-II upregulated) (Wang et al., 2019; Supplementary Table S6). Likewise, *P. fluorescens* SBW25 had a rhizosphere-expressed TTSS gene cluster showing similarities to pathogenicity-enhancing TTSS of *P. syringae* strain 61 and *E. amylovora*, although some of the genes of the TTSS system (needle formation) were missing in SBW25. Scientists reported that certain *Herbaspirillum* strains lack a T3SS system, whereas some contain a T3SS system (Straub et al., 2013). The absence of transposon elements adjacent to T3SS genes in *Herbaspirillum* HsSmR1 reduces the possibilities of the recent addition of T3SS genes through transposition. The T3SS flanking regions were conserved partially among *Herbaspirillum* strains, indicating the deletion of T3SS genes in certain *Herbaspirillum* strains like HfGSF30 (Pedrosa et al., 2011). Genome analysis of *Pantoea agglomerans* revealed that only four strains (PG734, P5, IG1, and 190) contain a partial or complete *hrc*/*hrp* gene cluster, and PG734 is the only strain containing a complete *hrc*/*hrp* gene cluster, but it is pathogenic. This study concluded that, in the absence of complete T3SS, P5 would be incapable of eliciting defense responses and could not convert into a pathogen, meaning it is considered as a safe biofertilizer (Shariati J et al., 2017). Further, a beneficial strain of *Pseudomonas syringae* strain 260-02 differs from its pathogenic counterpart, as it does not secrete toxins, has more biocontrol activity, and lacks Type III effectors. It is referred to as a “pacifist pathogen.” In the course of evolution, these genes became pseudogenes or got lost due to methylation events or HGT, indicating genome plasticity leading to contrasting behavior of pathogenic and beneficial strains (Passera et al., 2019). Similar to *P. syringae*, *Xanthomonas*, a well-recognized pathogen, lacked Hrp T3SS and TALEs required for pathogenesis and showed no symptoms in host plants like wheat, barley, and ryegrass (Li et al., 2020). This infers that evolutionary events led to variation in rhizobia, and HGT also incorporated T3SS system into phylogenetically unrelated bacteria (Nelson and Sadowsky, 2015; Hotinger et al., 2021), but the gene transfer was either incomplete or genes were lost after the acquisition by the various strains, which may have led to the formation of disarmed pathogens (Rezzonico et al., 2005).

*Rhizobium* utilizes Nod factors and their receptors (NFRs) for symbiotic association with leguminous plants (Figure 1B). Other effectors might be involved in mounting defense responses and may even increase or decrease symbiotic and colonization capacity (Clúa et al., 2018). There are various T3SS effectors involved in modulating host immunity, like NopD, which is secreted via T3SS and is homologous to the XopD of *Xanthomonas campestris*. More effectors involved in modulating host immunity include Blr1693 and Bl18244 of *Bradyrhizobium japonicum* (bja), and Msi059 of *M. loti,* which are secreted via the Type 4 secretion system (Nelson and Sadowsky, 2015). Flavonoids and NodD are extensively involved in regulating T3SS, its effectors, and expression of nod gene, stimulates ttsI expression encoding a regulator which interacts with conserved promoter elements (tts boxes-ranging from 7 to 30 in different rhizobial species) of secretion machinery and effectors (Wassem et al., 2008). In rhizobia, nodulation protein NopM plays a role in MAMP-triggered immunity by acting as an novel E3 ubiquitin ligase (NEL ligase), which activates flagellin sensitive 2 (flg22-induced) reactive oxygen species bursts in host plant *Nicotiana benthamiana* (Supplementary Table S6). Similarly, *Sinorhizobium* (*Ensifer*) *fredii* HH103 T3SS (secretes NopL, NopM, and NopP) suppresses defense responses for initiating nodulation in soybean. Even *Pseudomonas fluorescens* strain Q8r1-96 carries three T3SS effector proteins, RopM, RopAA (similar to *P. syringae* homologs HopM1 and HopAA1-1), and RopB (no homology), which have a role in suppressing PTI and ETI immune responses, suggesting the role of T3SS in immune suppression and host colonization.

**Figure 2:**
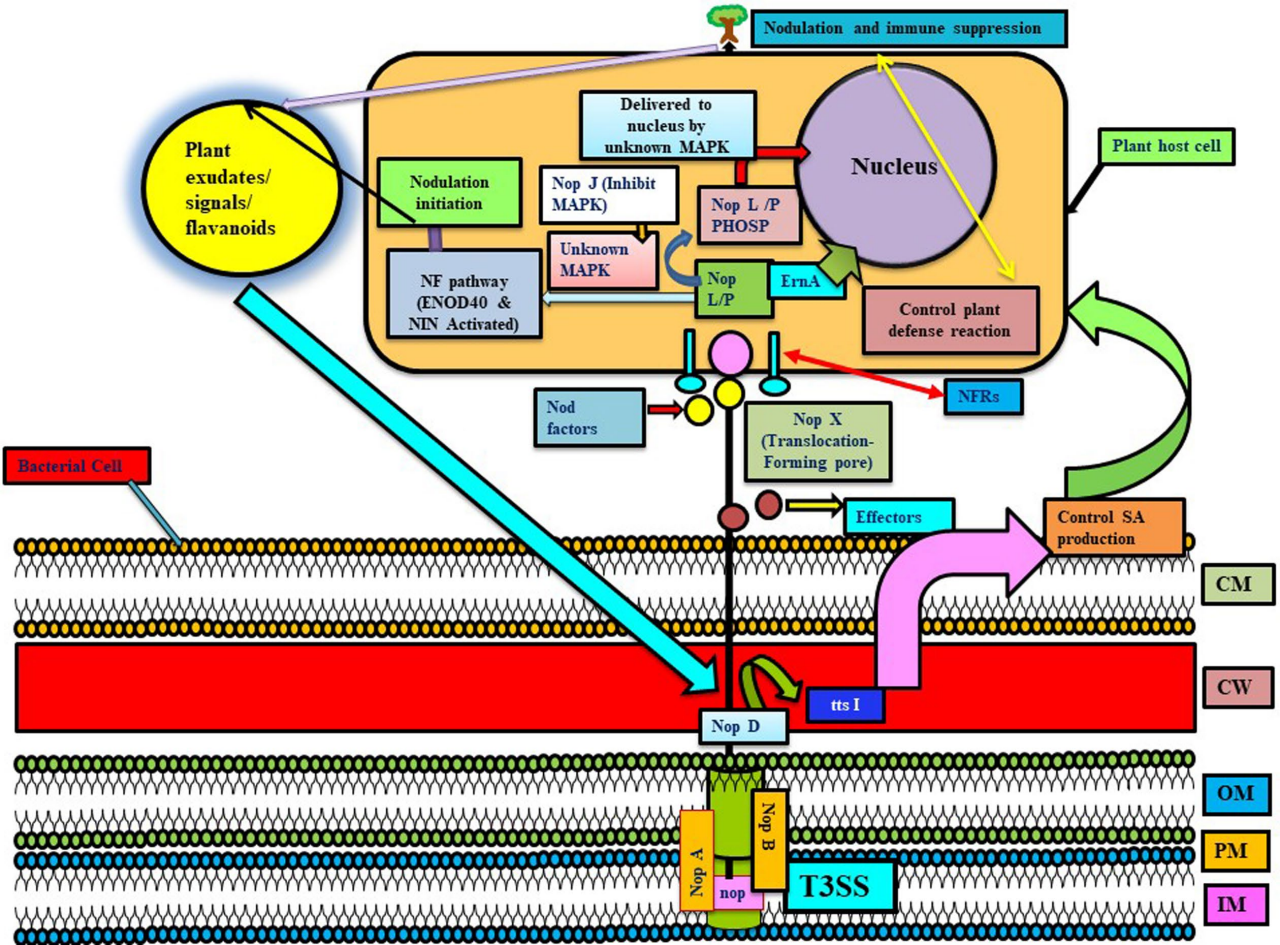
Represents the T3SS system in different Rhizobial species involved in the transfer of nop proteins. NopA and NopB are involved in the formation of T3SS-pilus, and NopX helps in translocating proteins in the host interior by forming a pore. In this, Nops are received by NF receptors, and then, NopL/M are phosphorylated by unknown MAPK and delivered to the nucleus via unknown MAPK (NopJ are involved in inactivating MAPK, thus they may have an inhibitory role in nodulation). These Nops induce early nodulation genes like ENOD40 and NIN and, thus, form an infection thread to promote nodulation. Further, Nops are involved in host immune suppression. NopD and ttsI are involved in regulating the T3SS system, whereas ttsI further controls the production of salicylic acid and possibly other defense responses in host plants, thus promoting the nodulation process and colonization. ErnA: an effector molecule targeting plant nucleus (unknown function). Here, various NOPs have different functions like NodO (signaling for nodulation), NopL (inducing plant immune response), NopM (ubiquitination), NopP (phosphorylated by plant kinases), NopT (cysteine protease activity), NopJ (inactivating MAP kinases), etc. (reviewed in Lucke et al., 2020) (adapted from Okazaki et al., 2013).

T3SS have also shown to play a role in nodule initiation and nodule organogenesis, established through various studies. Like genome sequencing of Sinorhizobial strains (HH103, USDA257, and NGR234) revealed that NopA and NopB (form T3SS-pilus of NGR234), whereas other Nop factors like NopL, NopP, and NopC are rhizobia specific (López-Baena et al., 2016). Different *Sinorhizobial* strains nodulate a wide variety of soybean, like USDA257 nodulates soybean (only Asiatic variety), HH103 (both Asiatic and American variety), and NGR234 (No variety), while USDA257 mutants are not able to secrete nop factors for nodulating American soybean variety (López-Baena et al., 2016). USDA257 showed more upregulation of genes involved in forming infection threads and nodules (i.e., *NIN* and *ENOD40* genes) than T3SS mutant strain (Jiménez-Guerrero et al., 2015). It has been observed that HH103 strain stimulates nodule formation due to extra copies of T3SS regulator ttsI in a *nodD1* mutant (not synthesizing NF and Nops), while in the T3SS mutant, such induction is not observed. Further studies revealed that impairment of ttsI enhanced the level of salicylic acid in soybean roots and leaves in uninoculated control plants and parental strain inoculated plants. Thus, it infers that HH103 T3SS is responsible for nodulation by controlling the SA induction and preventing dwarfism due to the overproduction of salicylic acid (not ethylene) (Jiménez-Guerrero et al., 2015; Figure 2).

*Bradyrhizobium elkanii* T3SS plays a role in symbiosis by activating host nodulation signaling when NFs and NFRs are absent (Okazaki et al., 2013). *M. loti* MAFF303099 and *Bradyrhizobium elkanii* secrete various flavonoid-inducible effector proteins, pathogen effectors, and molecules, hampering the plant metabolism via Type III systems (Sánchez et al., 2009; Okazaki et al., 2013). This study also infers that *B. elkanii* activates host symbiosis signaling for producing infection by changing the pathogenic system. *B. japonicum* secretes homologs of NopP, unknown proteins like NopE1 and NopE2, GunA2, and probably NopL, NopM, and NopT homologs via the Type III system (Zehner et al., 2008). Self-cleavage of NopE1 and NopE2 in the presence of calcium leads to stimulation of soybean nodulation and suppression in mung bean nodulation (Wenzel et al., 2010). T3SS mutation in *Bradyrhizobium* sp. DOA9 strain abolishes the capacity of the strain to interact symbiotically with legumes, unlike rice (Songwattana et al., 2017). Recently, researchers described how ErnA acts as a Bradyrhizobial effector molecule secreted by T3SS targeting plant nucleus, which may bind to the nucleic acid of the host cell and is found to play a role in nodulation (Teulet et al., 2019). Three analyzed nitrogen-fixing *Ensifer fredii* strains have type I, II, III, and IV SS, but *E. alkalisoli* YIC4027 do not contain type II, V, or VI SS genes. In this, T3SS-I is situated on the symbiotic plasmid pYIC4027a, while T3SS-II is present on the chromosome. T3SS-I of *E. fredii* plays a role in host-specific nodulation, exporting effector proteins, and stimulating immune responses in host plants. *E. fredii* strain YIC4027 lacks *nopA* and rhizobial effector genes, indicating the presence of a dysfunctional T3SS with a limited host range (Dang et al., 2019). In an incompatible interaction of USDA61 and soybean BARC2 cultivar., cysteine protease (homologous to xopD) of USDA61 interacts with Rj4 (R-protein) of soyabean-enhanced hydrogen peroxide and salicylic acid accumulation and further led to inducing defense genes during early phases of interaction. So, rhizobia effector proteins act as double-edged swords in host microbe recognition. During compatible interaction, bacteria effectors induce nodulation by lowering host defense responses, but when incompatible R proteins hosts recognize bacterial effectors, ETI are triggered and the nodulation process is hampered (Clúa et al., 2018).

Several studies indicate the importance of T3SS in causing infection and colonization in host plants. For example, *Bradyrhizobium* sp. SUTN9-2 contains T3SS (*rhcJ*) and T4SS (*virD4*), which play a role in infecting rice and leguminous crop. The T3SS and T4SS system and pectinesterase (peces) genes might be involved in the early steps of infection in rice and overcoming plant defense (Piromyou et al., 2015). Similar to *Burkholderia phytofirmans* PsJN and *Herbaspirillum seropedicae* strain SmR1, *Bradyrhizobium* sp. SUTN9-2 also contains T3SS (*rhcJ*) genes for endophytic colonization in host plants (Piromyou et al., 2015). In addition, *P. fluorescens* strains like WH6, KD, Q8r1-96, and BBc6R8 contain a T3SS system. In a Mycorrhiza Helper Bacterium (MHB), *P. fluorescens* strain BBc6R8 T3SS mutants were found to be unable to promote mycorrhization (Cusano et al., 2011). T3SS gene clusters (tts) are present in *Pseudomonas* strains WCS417 and WCS374 in 26-kb and 18-kb clusters, respectively, showing significant resemblance with the *hrp*/*hrc* cluster of phytopathogenic *Pseudomonas species*. PGPR *Pseudomonas* strain WCS358 genome lacked a tts cluster and other genes encoding T3SS components, while WCS417 contained RopE of the *AvrE* family of effectors near to *rspL*, regulating the tts cluster (Berendsen et al., 2015). Similar to *P. fluorescens* SBW25, T3SS of WCS374 and WCS417 are regulated by RspL (Zboralski et al., 2022). Unlike WCS374, WCS417 lacked needle-forming proteins, and proteins related to effector translocation in T3SS were absent in both (Stringlis et al., 2019). Similar to *P. fluorescens* strain BBc6R8, *P. fluorescens* C7R12 T3SS mutant was unable to induce root colonization by AMF in the *Medicago truncatula* (Viollet et al., 2017). Mutational changes in *H. rubrisulbalbicans hrpE* and *hrcN* gene showed failure in causing infection in the sugarcane lesions on *Vigna unguiculata* leaves. Mutation in beneficial or symptomless *H. rubrisubalbicans* led to abolishing entry into rice and maize, suggesting a change in the function of T3SS genes according to the host (Schmidt et al., 2012).

T3SS also play a role in biocontrol activity. PGPR *Pseudomonas fluorescens* 2P24 T3SS gene cluster *rsp/rsc* showed a significant resemblance to the *P. syringae hrp/hrc* cluster. But it lacks the regulator genes *hrpR*, *hrpK1,* and *hrpH* that play a role in protein exporting. The results showed that innate immunity generated against Rsp would help explain the concept of immunity and resistance developed by PGPR in host plants. Mutation of the *rscC* gene did not affect the biocontrol property, although it abolished the secretion of effectors (Liu et al., 2016). Recently, it has been shown that mutation in the transcriptional regulators (hilA and invF) of 2P24 T3SS led to decrement in resistance against *Fusarium graminearum* (2P241invF), increased biofilm formation (2P241hilA), reduced chemotactic responses (2P241hilA, 2P241invF, and 2P241invE-C), and reduced reactive oxygen species’ production (2P241invE-C) more than wild type strain 2P24 (Wang et al., 2021). *P. fluorescens* strains WH6 and KD contain a complete and functional T3SS showing homology with the T3SS region of *P. syringae.* Mutation of *hrcV* (gene of TTSS system) strongly hampered the biocontrol capacity of *P. fluorescens* KD against *P. ultimum,* causing damping-off in cucumber. The mutation in *hrcV* reduced the inhibition of pectinase production in *P. ultimum* by *P. fluorescens* KD. As pectinase is a key virulence factor of *P. ultimum*, strain KD reduced the infection capability of *P. ultimum* in plants by reducing pectinase production in *P. ultimum* (Rezzonico et al., 2005). However, mutational studies revealed that T3SS-deficient mutants have no loss in the colonization and biocontrol capacity, indicating a lack of any direct role of T3SS in the biocontrol and colonization process (Mavrodi et al., 2011).

Functional studies in different symbiotic strains of different rhizobial species strains including *M. loti, R. etli, B. japonicum, B. elkanii, B.* sp. SUTN9 *S. fredii, S.* sp. HH103, USDA257, *Ensifer fredii* and *E. alkalisoli* indeed demonstrated the role of T3SS in induction of genes responsible for infection thread formation, suppression of defense response to initiate nodulation, and control of salicylic acid accumulation. Hence, T3SS plays a critical role in the initiation and establishment of the legume-rhizobia interaction. However, despite the presence of T3SS genes in several, such as *P. fluourescens, P.* sp. WCS374, *H.* sp. HsSmR1, *H. rubrisubulbicans,* and *P. agglomerans,* the direct role of TS33 genes in colonization, establishment, and biocontrol has not been demonstrated. Though the sporadic reports hints toward possible roles of T3SS in PGPR-plant interaction, with the exception of rhizobial strains, fully functional T3SS seems to be more involved in plant-pathogen interaction than beneficial interactions.

### 2.6 Type 4 secretion system

T4SSs were observed more commonly in endophytes than in rhizospheric bacteria. T4SS, like F-type (virB and trb families) and the P-type cpa/tab/pli system, are present mainly in two Rhizobial families. In this, virB/trb genes are responsible for virulence and conjugal transfer, while P-type works as flagella proteins. T4SS of *Rhizobium tropici* PRF 81 contains various genes encoding proteins that play a role in the conjugation-transfer system, exhibiting homology to *R. etli* and *S. meliloti* (Figure 1B). T4SS of *Rhizobia* is involved in the conjugative transfer of symbiotic plasmids such as *Rhizobium etli* and *S. meliloti*. Like T3 and T4 effectors of phytopathogens, two effectors, Msi059 and Msi061 of *Mesorhizobium loti* strain R7A, are also secreted through T4SS (Nguyen et al., 2017). Msi061 could have a role in degradation via proteasomes through protein ubiquitination, which is quite similar to VirF of *Agrobacterium tumefaciens*. Msi059, meanwhile, might exhibit similarity to a T3 effector protein XopD of *X. campestris* pv vesicatoria, which has plant-specific SUMO substrate protease activity (Nguyen et al., 2017). Symbiotic competence of *M. loti* decreased for *Lotus japonicus* due to loss of T4SS, but *M. loti* can make nodules on *Leucaena leucocephala*, although it is incapable of causing infection. T4SS mutants of rhizobia like *S. meliloti* did not show any decrement in symbiotic activity; therefore, the significance of T4SS and their putative effectors in symbiosis are still in question (Santoyo et al., 2021). Unlike Rl 3841, Rl Norway pertains to a different T4SS encoded in the pRLN1 plasmid and is found to be responsible for transferring proteins rather than DNA, because VirD2 relaxase is absent in it (Liang et al., 2018; Supplementary Table S7). It has been observed that *M. loti* R7A and MAFF303099 T4SS stabilize symbiosis by controlling the nodulation rate, while *E. meliloti* 1021 was found to be important in conjugation but not for symbiosis. T4SS-related genes are present on plasmids, even in multiple numbers in various species, suggesting the possibility of multiple events of HGT (Carvalho et al., 2010). T4SS is absent in most of the *Herbaspirillum* strains; still, some *Herbaspirillum* strains like AzospB510, Kp342, and GdPAI5 contain T4SS genes (Straub et al., 2013). The *Pseudomonas putida* strain IsoF utilizes the type IVB secretion system (T4BSS) for bacterial competition by delivering toxic effectors into rhizospheric- and plant-associated bacteria through contact-dependent killing. Through horizontal gene transfer, the kib gene cluster responsible for encoding T4BSS is acquired by *P. putida*, and it also helps in invading existing biofilms, providing better persistence in the environment and preventing phytopathogen *Ralstonia solanacearum* via the T4BSS system (Purtschert-Montenegro et al., 2022). However, a recent report suggests the role of the type IVA secretion system (T4ASS) of *Lysobacter enzymogenes* to be producing a T4ASS effector protein Le1519 (LtaE: Lysobacter T4E triggering antifungal effects), which inhibits the repressor of antifungal antibiotic 2,4-DAPG and initiates the production of 2,4-DAPG by *Pseudomonas protegens*. Thus, the T4ASS of one bacterium enables the production of antifungal 2,4-DAPG in another and helps in reducing the infection of fungus *Rhizoctonia solani* in soyabean plants, which is a unique example of interspecies and interkingdom interaction (Wang et al., 2023).

The role of the Type IV secretion system is not well understood in the plant-bacteria interaction, although these few studies revealed its role in microbial competition (Dias et al., 2019), infection, symbiotic competence (Nguyen et al., 2017; Guo et al., 2022), protein transfer (Liang et al., 2018), biocontrol, and mutualistic interaction as well (Wang et al., 2023).

### 2.7 Type 5 secretion system

Type V secretion systems are much smaller, differ in complexity, and are comprised of a single polypeptide and a multi-protein complex spanning several membranes (Meuskens et al., 2019). Three orthologs, autA, autB, and autC of Type V, are present in all the fourteen Rhizobiales studied previously (Black et al., 2012). Type V orthologs are also identified in non-symbiotic *Mesorhizobium* but contain virulence or infection potential. Autotransporter proteins secrete proteins into the periplasm via GEP using an insert into the outer membrane. C-terminal domains are inserted in the OM and initiate the export of the N-terminal region outside the cell (Meuskens et al., 2019). Near the C-terminus, certain proteins containing a distinctive autotransporter domain and similar kinds of proteins were observed in *R. leguminosarum* bv. viciae strain 3,841. However, mutation of these autotransporter genes did not affect the symbiotic interaction capacity of *R. leguminosarum* (Krehenbrink and Downie, 2008; Figure 1B). Rlv 3841 contains three auto-transporters, while a two-partner system was absent. Rl Norway, on the other hand, harbors two T5SS auto-transporters and one two-partner system, with filamentous hemagglutinin acting as the putative cargo protein (Liang et al., 2018; Supplementary Table S8).

In plant-beneficial *Pseudomonas* WCS358, WCS374, and WCS417, strains, 2, 10, and 5 T5aSS autotransporters are located, respectively. However, the functions of these autotransporters are not well defined, but they show homology to autotransporters of *P. aeruginosa* PAO1, having a role in triggering host defense, peptidase activity, biofilm formation, and motility. In WCS358, WCS374, and WCS417 orthologs containing the POTRA (polypeptide transport-associated) domain, substrate protein TpsA is translocated via T5SS. TpsAs enable WCS strains to colonize successfully in plant roots by inhibiting their competitors. Recently, a single ortholog of patatin-like protein PlpD showing lipolytic activity against plant pathogens was found in each of the WCS genomes (Berendsen et al., 2015).

There are three putative T5SS present in *Pseudomonas* UW4 (Duan et al., 2013). Out of them, one AT showing esterase activity has been reported to have a major effect on twitching, swarming, and swimming motilities of *P. aeruginosa*. In *Pseudomonas* UW4, two putative ATs code for an outer membrane autotransporter and an extracellular serine protease. Unlike other Pseudomonads, UW4 lacked any of the two-partner secretion systems (TPS).

On genome comparison of several *Pseudomonas putida*, only strain W619 contains a ndvB gene producing β-(1,2)-glucan, a putative adhesin, a surface-adhesion calcium-binding outer membrane-like protein similar to biofilm-forming protein LapA, and two putative genes for adhesion, which code for autotransporter proteins having a pectin/lyase/pertactin domain (Wu et al., 2011).

Approximately thirty clusters of three T5SS types (T5aSS, T5bSS, and T5cSS) were observed in the three *P. kururiensis* genomes. The two-partner system (TPS) of *B. thailandensis* helps in evocating competitors and enhances interaction with bacteria (Garcia et al., 2016). Although the role of TPS in strain KP23T is not well understood, the presence of several T5SS clusters indicates implications in the plant colonization process. The current understanding suggests the role of T5SS in motility and antagonism to competing bacteria and fungi, helping the PGPR in colonizing the host plants. The inputs of T5SS in the plant-beneficial bacterial interaction have yet to be understood and investigated (Dias et al., 2019).

### 2.8 Type 6 secretion system

T6SSs of Gram-negative bacteria are contractile nanomachines comprised of core components (TssA-M), and the transport mechanism is well-reviewed. Implications of T6SS in root nodulation are well documented in symbionts such as *Rhizobium* spp., *Mesorhizobbium* spp., and *Sinorhizobium* spp. This could be clarified by an example, i.e., *Rhizobium leguminosarum* bv. trifolii possesses a novel gene cluster (RbsB (ribose binding protein) 27-kDa protein exported by T6SS and *vas* (virulence secretion association) genes) encoding proteins that inhibit nodule formation and are involved in host interaction and nitrogen fixation (Hersch et al., 2020). Mutation of the imp (impaired in nodulation) locus (a T6SS locus) secretion system of *R. leguminosarum* fixed nitrogen in a non-host plant pea (Hersch et al., 2020; Figure 1B). Deletion of the gene locus of T6SS in *R. leguminosarum* enabled nitrogen-fixing capability in pea, which is generally a non-host plant. While proteins excreted by this system had homology to Rbs proteins of other bacteria, mutations were done to understand the importance of these proteins in nitrogen fixation in pea (Hersch et al., 2020; Supplementary Table S9).

A comparative analysis was conducted on a set of ten *Neorhizobium galegae* strains, revealing that six of them exhibited the T4SS type 4 secretion system, three strains possessed the T4SS type I secretion system, and six strains were identified as having the T6SS (the type I and type IV systems mentioned here are known to be the quorum sensing (QS)-regulated conjugation system). This study revealed that T4SS and T6SS might not be involved in the symbiosis of *N. galegae* (Österman et al., 2015) and their exact role has yet to be identified.

Further, biocontrol activity in a variety of strains of rhizospheric bacteria in the *Pseudomonas* genus might be due to T6SS. *Pseudomonas fluorescens* MFE01 strain can inhibit *Pectobacterium atrosepticum*, which causes tuber soft rot in potato tubers. This MFE01 strain has three different genes (*hcp1*, *hcp2*, *hcp3*) related to T6SS. T6SSs harboring Hcp2 or Hcp3 show antibacterial activity, while Hcp1 is responsible for bacterial motility (Decoin et al., 2014). Another finding reported that MFE01 *hcp2* is responsible for killing *Pectobacterium atrosepticum*, which is mediated by T6SS effectors targeting the peptidoglycan of Gram-negative bacteria (Decoin et al., 2014). Similarly, *P. fluorescens* Pf29Arp harbors four complete T6SSs gene clusters (cluster I, II, III, and IV), along with the T3SS protein secretion system. This strain has a biocontrol effect on the disease-causing pathogenic fungus *Gaeumannomyces graminis* var. tritici, and studies showed that T6SS genes showed more expression in fungus-inhabiting roots. Despite the lack of specific elucidation regarding the precise function of T6SS-mediated protection, its presence suggests a potential involvement of T6SS in facilitating the adaptability of Pf29Arp in varying habitats (Marchi et al., 2013). Studies revealed that the production of iron-chelating siderophores, especially peptidic siderophores (e.g., pyoverdin) by *P. taiwanensis,* might be the reason for the inhibition of *Xanthomonas oryzae* pv. Oryzae. It has been reported that pyoverdin accumulated in the periplasm of *P. taiwanensis* T6SS mutants, so that this T6SS substrate can be transferred from the periplasm. However, it requires experimental procedures to check the direct secretion of pyoverdin via T6SS, but it suggests that T6SS might be involved in the regulation of pyoverdine secretion (Chen et al., 2016).

In *Pseudomonas* W619, a T6SS-associated gene cluster located in genomic region 20 showed dissimilarity with different *P. putida* strains (KT2440, F1 and GB-1) (Wu et al., 2011). *P. putida* KT2440 can erode a wide range of phytopathogens like *Xanthomonas campestris*, *P. syringae*, *A. tumefaciens,* and *Pectobacterium carotovorum*, mainly with the help of T6SS. Studies implicated the role of T6SS in biocontrol by mutational studies and found that the KT2440 T6SS mutant strain cannot protect *Nicotiana benthamiana* from leaf necrosis like wild strain (Duan et al., 2013).

**Figure 3:**
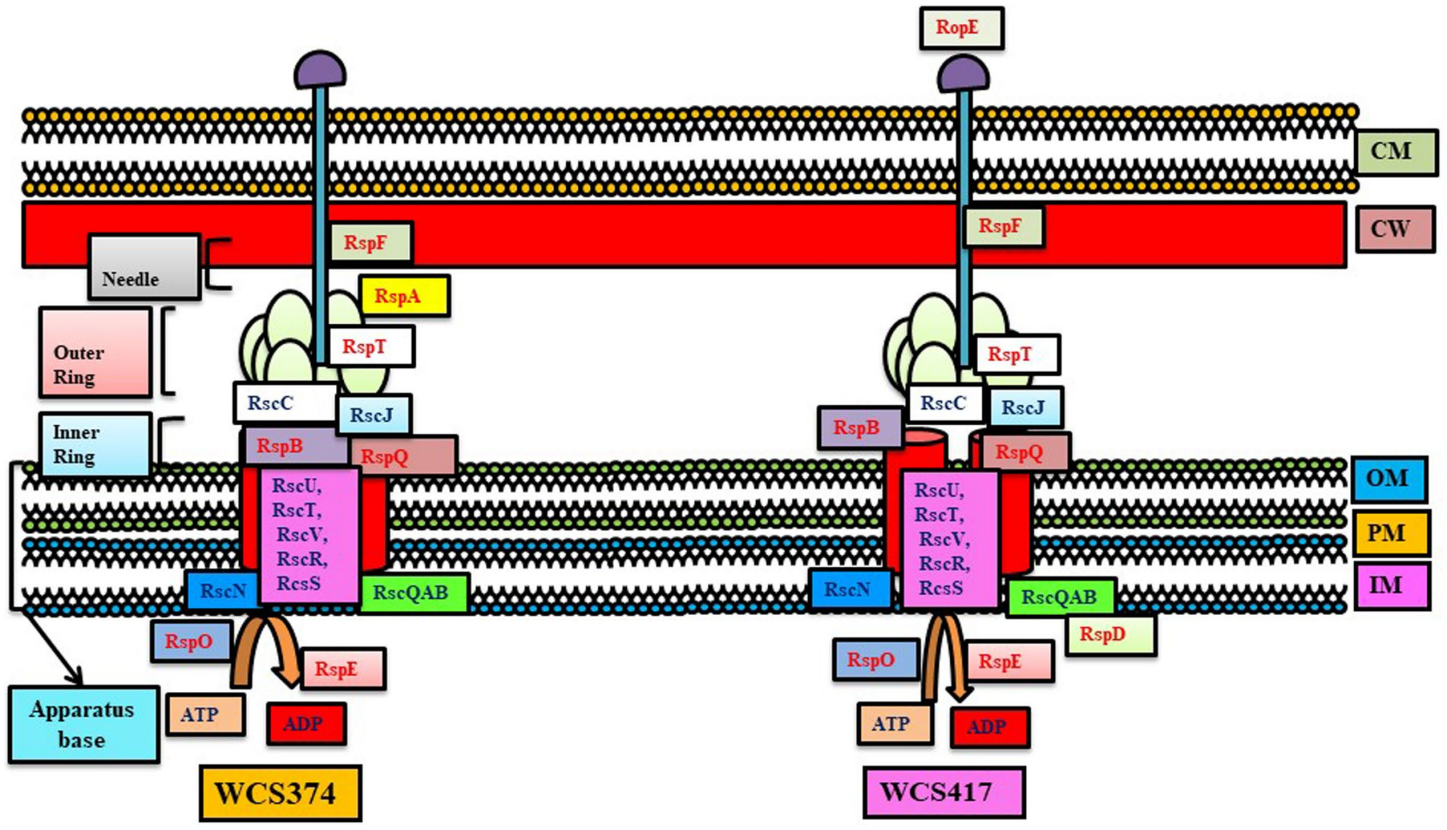
T3SS system in *Pseudomonas* WCS374 and WCS417. The conserved genes are written with black letters and non-conserved with red letters (adapted from Stringlis et al., 2019).

Various studies suggest the importance of T6SS in the colonization of PGPR into host plants. Genomes of Pseudomonads WCS417 and WCS374 have two T6SS loci each, while WCS358 contains a single locus (Berendsen et al., 2015). Recently, scientists reported antibacterial Hyde1 and/or Hyde2 as a new T6SS effector family, which clears competitive bacteria via T6SS and increases the persistence of *Acidovorax citrulli* on its watermelon host. *Pseudomonas protegens* contain T6SS apparatus for killing cabbage pest *Pieris brassicae* through T6SS spikes (VgrG1a and VgrG1b) and associated effectors (RhsA and Ghh1) and helping in host colonization and pathogenesis (Vacheron et al., 2019; Figure 3).

Diazotrophic *Azoarcus oleaius* T6SS has two putative T6SS gene clusters, tss-1 (upregulated under nitrogen-fixing condition) and tss-2 (constitutively expressed), having homology with T6SS of *P. aeruginosa*. Various genes related to threonine-phosphorylation pathway-dependent and -independent regulatory pathways are present in the *tss-2* gene cluster. These two pathways post-translationally regulate T6SS-2 but not T6SS-1 activity; thus, it infers that both pathways lack any kind of regulatory cross-talk between them. This study revealed that, unlike T6SS-1, T6SS-2 is more conserved, showing a resemblance with most of the *P. aeruginosa* strains. This suggests that T6SS-2 has a more conserved function, while the function of T6SS-1 is quite specific for *A. olearius* BH72, and during nitrogen fixation, T6SS-1 genes get upregulated, forming related cellular proteins but still lacking T6SS-1 activity. This could potentially be attributed to the lack of *tssH*, or alternatively, it could be mediated by *tss-1* gene cluster homologs or by an alternative mechanism (Jiang et al., 2019).

**Figure 4:**
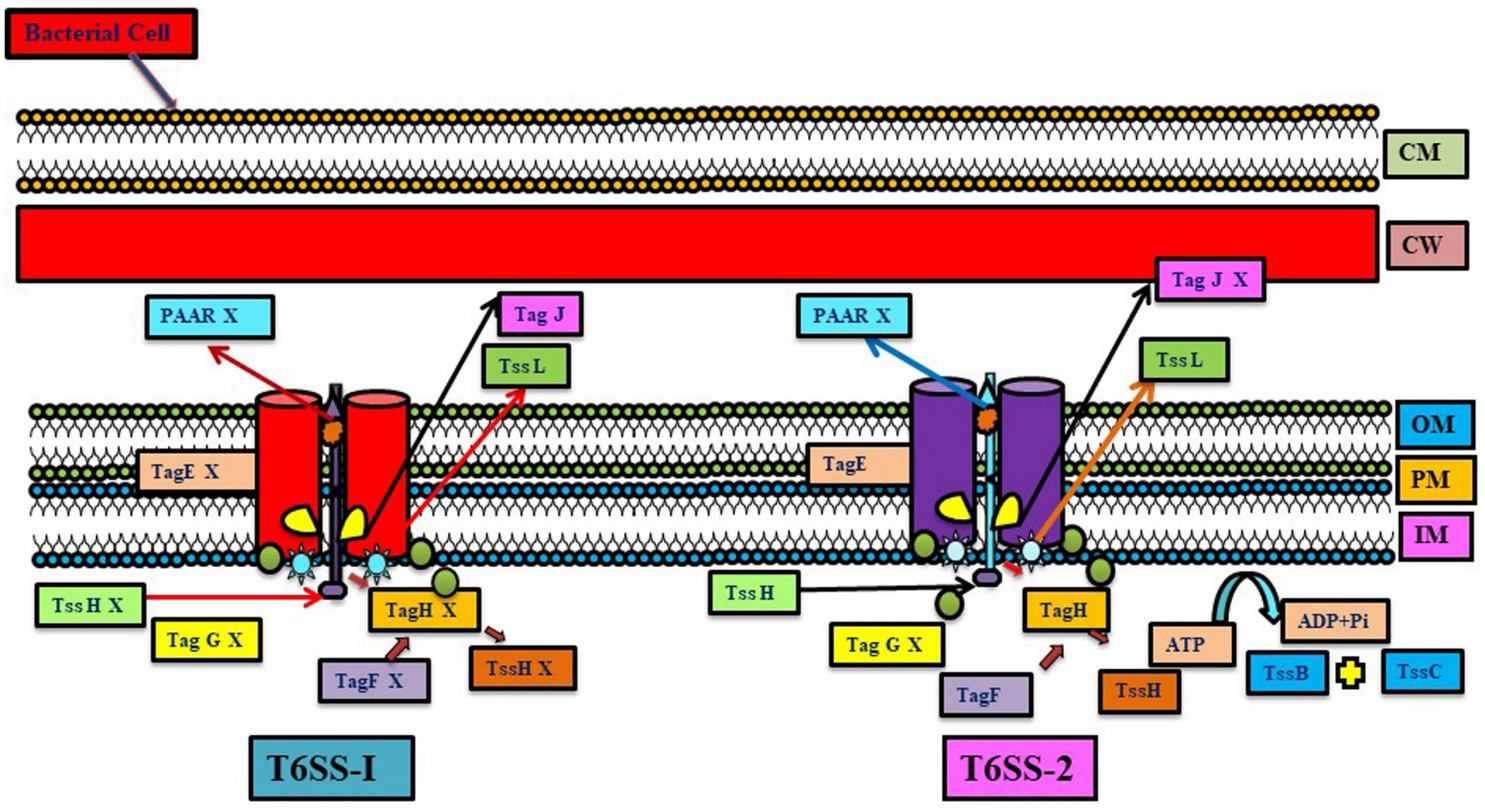
Representation of the difference between two T6SS systems (T6SS-1 and T6SS-2) present in *Azoarcus oleaius*. Different genes present in the system are depicted (Gene X shows the absence of that gene). T6SS gene cluster tss-1 (upregulated under Nitrogen fixing condition) and tss-2 (constitutively expressed) have homology with T6SS of *P. aeruginosa* (adapted from Jiang et al., 2019).

Unlike the above studies, T6SS diminishes endophytic colonization, as observed in the T6SS mutant of *Azoarcus* sp. BH72. This mutant showed more colonization than the wild-type strain, suggesting that certain effectors translocated by T6SS may elicit a local immune response and can limit colonization (Shidore et al., 2012). Mutational analysis in *Azoarcus* sp. strain BH72 of various differentially controlled genes encoding minor pilin PilX, signal transduction proteins and a serine–threonine kinase of the T6SS system was carried out, which indicated their importance for establishment in host plants. *Azoarcus* sp. CIB has different secretion systems like T1SS, T2SS, T4SS, and T6SS systems. The T6SS system is also present in the genomic island XI of *Azoarcus* sp. CIB (Martín-Moldes et al., 2015; Figure 4). Similarly, a *tss* mutant *Enterobacter* sp. J49 affecting T6SS functionality deaccelerated the epiphytic and endophytic colonization rate in comparison to the wild strain, suggesting the role of T6SS in the colonization process (Lucero et al., 2022).

Most *Herbaspirillum* strains and grass endophytes have T6SS except for the GdPAI5, HIP6-12, and HJC206 strain. *H. rubrisubalbicans* was initially identified as a phytopathogen causing mottled stripe disease in *Saccharum officinarum*. But *H. rubrisubalbicans* is also capable of associating with different host plants without causing any pathogenic effect (Andrés-Barrao et al., 2017).

A comparative genomic analysis was conducted on three *Paraburkholderia kururiensis* genomes, revealing the presence of multiple gene clusters of T6SS in strains KP23T, M130, and ATSB13T (Dias et al., 2019). *Azospirillum brasilense* Az39 has two complete sets of genes related to type VI secretion systems (T6SS), where T6SS1 is reported to be stimulated by indole-3-acetic acid (IAA) phytohormone. *A. brasilense* T6SS was found to be involved in lipid production, carbohydrates, photosynthetic pigments, attachment, IAA production, and bio-control against bacterial pathogens in microalgae *Chlorella sorokiniana* (Cassan et al., 2021). Three T6SS loci (T6SS-1, -2, and –3) are located in different *P. ananatis* strains. Among T6SS loci, it has been observed that the T6SS-1 locus is prevalent in a majority of *P. ananatis* strains. Conversely, the T6SS-2 locus has been documented exclusively in pathogenic strains, while the T6SS-3 locus has been identified in only a limited number of strains. Since T6SS-1 and T6SS-2 are observed in both pathogenic and non-pathogenic strains, it can be inferred that they do not serve as exclusive pathogenic determinants. However, T6SS could potentially play a role in competition, fitness, or niche adaptation, while other studies suggest the role of T6SS to be in nodulation, biocontrol, motility, killing, and antagonism against bacterial and fungal pathogens (Sheibani-Tezerji et al., 2015; Lu et al., 2022; He et al., 2023). It has also been shown to induce production of phytohormones and siderophores, which are known to induce plant growth. Hence, T6SS can have beneficial effects on the growth of host plants.

### 2.9 Type 7 secretion system

Earlier, the T7SS secretion system was mostly reported in Gram positive bacteria and found to be conserved in *Bacillus* species (Abdallah et al., 2007; Lu et al., 2022; He et al., 2023). Although it has now been reported in some *Pseudomonas* species (Supplementary Table S1). In *Bacillus* species, T7SS is encoded by yuk/yue operon secreting YukE showing homology with EsxA of *Mycobacterium*. The function of T7SS is not fully understood yet, but a recent report suggests the role of *Bacillus velezensis* SQR9 T7SS in niche colonization as the knock-out mutant of T7SS and YukE colonization rate was decreased. This YukE might be involved in changing membrane permeability and further lead to iron loss at the early stage of infection. Thus, T7SS is important for niche colonization in mutualistic interactions with host plant (Liu et al., 2023).

### 2.10 Type 9 secretion system

Type IX secretion system (T9SS) is exclusively found in the Bacteroides phylum (Abby et al., 2016), and its genus *Flavobacterium* is used for understanding gliding motility in which motility adhesions are exported via T9SS. Currently, the location of T9SS components is well known, but their exact functions are still not very clear (Gorasia et al., 2020).

*Flavobacterium* is highly abundant in the rhizosphere, possibly due to the secretion of extracellular enzymes such as peptidases, chitinases, and several glycoside hydrolases involved in a plant’s metabolic reactions and host development and resistance toward pathogens. It also contains a *Bacteroidetes-*specific gliding-motility complex, which is linked with the T9SS system responsible for secreting extracellular hydrolytic enzymes like peptidases, chitinases, and protease (McBride and Zhu, 2013). A gliding-motility-associated lipoprotein, GldJ, is responsible for stabilizing the interaction between T9SS and gliding-motility complex, and on deletion of *GldJ*, gliding-motility and chitinase secretion are abolished. Apart from this, motility proteins SprB and RemA are also secreted by the T9SS system, which helps in motility, colonization, and elicitation of plant defense responses (Kolton et al., 2016).

In the colonization of bacteria, motility is an extra advantage for rhizosphere competence. It has been reported that the gliding-motility-T9SS complex provides a competitive advantage for flavobacterial surface attachment, seed adhesion, plant root colonization, and in persistence of flavobacteria after surface attachment (Shrivastava et al., 2015). Plant exudates or mucilage are responsible for enforcing the flavobacteria movement from soil toward the roots by the activation of the gliding motility-T9SS complex. It has been revealed by previous studies that *Flavobacterium* strains have a wide array of genes involved in carbohydrate catabolism like xylose, arabinose, pectin, and rhamnogalacturonan. In addition, many enzymes related to carbohydrate catabolism are secreted via T9SS. Mutational studies of T9SS unveiled that mutation significantly reduced the rhizosphere survival resistance to tomato pathogen *C. michiganensis*.

Hence, T9SS plays a pivotal function in enhancing rhizospheric competence, abundance, and resistance to plant disease. Nevertheless, it is necessary to gain a thorough comprehension of the precise function of the gliding-motility– T9SS complex in rhizosphere competence and plant protection. Gaining a complete understanding of the exact role of the gliding-motility– T9SS complex in rhizosphere competence and plant protection is still needed.

## 3 Conclusion

In the last few years, a lot of research has been done on various PGPRs but there still exists a large gap in understanding the role of the secretion system, their gene function, and their involvement in the colonization of PGPRs inside the host. Some studies indicate that Type I and Type II SS are observed in bacterial endophytes mostly, whereas Type III and Type IV SS are found to be missing in endophytes and show their presence in pathogenic bacteria. It has been observed that T5SS and T6SS systems are very common in endophytes and have several important roles during plant-microbe interactions. A comparative study among a large number of symbionts, endophytes, and phytopathogens indicated that T3SS systems are more common in symbionts and plant pathogens than in endophytes. However, endophytes have a significant number of T3SSs in comparison to soil bacteria. In contrast, endophytes have more T4SS and might be more involved in DNA conjugation and infecting hosts than rhizosphere bacteria. Even genes related to twitching motility required for adhering to the host and type I pilus assembly are more often present in endophytes than symbionts. Nevertheless, numerous unresolved questions remain about the exact structure and organization of different secretion systems in PGPRs, as well as the distinctions in their secretion systems compared to their phytopathogenic counterparts. However, various reports suggest that various PGPRs have similar genetic organization of secretion systems to pathogens. Many studies indicate the possibilities of various horizontal gene transfer events from pathogens and these being responsible for creating divergence and/or similarities among pathogenic and beneficial bacteria. Some also infer toward the evolution in the PGPRs itself, eliminating the chances of horizontal transfer. However, it is important to investigate how the secretion mechanism differs between the beneficial and pathogenic bacteria despite their comparable architecture. Additionally, it is necessary to explore whether a specific switch can transform the pathogen into a beneficial one based on certain conditions. Few studies reveal the importance of methylation and demethylation in governing the two diverse lifestyles, but this needs further investigations. Also, further investigation is required to understand how the secretion system regulates symbiosis and other significant metabolic events in beneficial bacteria.

Various mutational, metabolomic, transcriptomic, and genome-based studies can be helpful in solving the puzzle, and the role of the secretion system in plant-beneficial bacteria would further enhance the required knowledge of communication between the host and the PGPRs.

## Author contributions

GG: Formal analysis, Investigation, Methodology, Writing – original draft, Writing – review & editing. PC: Data curation, Formal analysis, Project administration, Supervision, Writing – review & editing. PJ: Data curation, Methodology, Resources, Software, Writing – review & editing. RV: Data curation, Formal analysis, Software, Validation, Writing – review & editing. SS: Formal analysis, Investigation, Software, Validation, Writing – review & editing. VY: Data curation, Formal analysis, Resources, Software, Writing – review & editing. DS: Formal analysis, Funding acquisition, Project administration, Supervision, Writing – original draft, Writing – review & editing. AP: Conceptualization, Project administration, Supervision, Visualization, Writing – original draft, Writing – review & editing.
