# Supplementary material for: Secretory molecules from secretion systems fine-tune the host-beneficial bacteria (PGPRs) interaction
Source: Front Microbiol. 2024 Feb 26;15:1355750. doi: 10.3389/fmicb.2024.1355750 (PMC10925705; doi:10.3389/fmicb.2024.1355750)
Supplement: Supplementary file 1 [file Table_1.doc]

**Supplementary Table 1**

Different secretion systems present in PGPRs

| **PGPR** | **SEC** | **TAT** | **Type I** | **Type II** | **Type III** | **Type IV** | **Type V** | **Type VI** | **Type VII** | **Type VI II** | **Type IX** | **References** |
| --- | --- | --- | --- | --- | --- | --- | --- | --- | --- | --- | --- | --- |
| **Gram Positive** |  |  |  |  |  |  |  |  |  |  |  |  |
| ***Bacillus*** |  |  |  |  |  |  |  |  |  |  |  |  |
| *Bacillus amyloliquefaciens* FZB42 |  | + | + | + |  |  |  |  |  |  |  | Chen et al., 2007; Kierul et al. 2015 |
| *Bacillus megaterium* STB1 | + | + |  |  |  |  |  |  | + |  |  | Nascimento et al., 2020 |
| *Bacillus velezensis* 9D-6 | + | + |  |  |  |  |  |  | + |  |  | Grady et al. 2019 |
| *B. amyloliquefaciens* subsp. plantarum UCMB5113 | + | + |  |  |  |  |  |  |  |  |  | Niazi et al. 2014 |
| **Gram Negative** |  |  |  |  |  |  |  |  |  |  |  |  |
| ***Pseudomonas*** |  |  |  |  |  |  |  |  |  |  |  |  |
| *P. fluorescens* | + | + |  |  |  |  |  |  |  |  |  | Ma Q et al., 2003 |
| *Pseudomonas* WCS417 |  |  | + | + | + |  | + | + |  |  |  | Benderesen 2015 |
| *Pseudomonas* WCS374 |  |  | + | + | + |  | + | + |  |  |  | Benderesen 2015 |
| *Pseudomonas* WCS358 |  |  | + | + |  |  | + | + |  |  |  | Benderesen 2015 |
| *Pseudomonas* sp. UW4 | + | + | + | + | + |  | + | + |  |  |  | Duan et al. 2013 |
| *Pseudomonas* putida KT2440 |  |  | + | + |  |  |  | + |  |  |  | [Hinsa et al*.* 2003](javascript:;) |
| *Pseudomonas* putida W619 |  |  | + |  |  |  | + | + |  |  |  | Wu et al. 2010 |
| *Pseudomonas* *fluorescens* KD |  |  |  | + |  |  |  |  |  |  |  | Rezzonico et al. 2004 |
| *Pseudomonas chlororaphis* GP72 and *Pseudomonas fluorescens* Pf-5) and other two PGPRs (*Pseudomonas aeruginosa* M-18 and *Pseudomonas stutzeri* A1501 |  |  | + | + | + (Only M18) | + | + | + |  |  |  | Shen et al. 2013 |
| *Pseudomonas fluorescens* strains WH6, KD, Q8r1-96, and BBc6R8 |  |  |  |  | + |  |  |  |  |  |  | Rezzonico 2005 |
| *Pseudomonas* *fluorescens* C7R12 |  |  |  |  | + |  |  |  |  |  |  | *P. fluorescens* C7R12 |
| *Pseudomonas fluorescens* SBW25 |  |  |  |  | + |  |  |  |  |  |  | Rezzonico et al. 2004 |
| *Pseudomonas fluorescens* 2P24 |  |  |  |  | + |  |  |  |  |  |  | Liu et al. 2016 |
| *Pseudomonas syringa*e strain 260-02 |  |  | + |  | + | + | + | + | + |  |  | Passera et al. 2019 |
| *Pseudomonas viridiflava* CDRTc14 |  |  | + | + |  | + | + | + | + |  |  | Samad et al., 2017 |
| *Pseudomonas fluorescens* MFE01 |  |  |  |  |  |  |  | + |  |  |  | Decoin et al. 2014 |
| *Pseudomonas fluorescens* Pf29Arp |  |  |  |  |  |  |  | + |  |  |  | Marchi et al., 2013 |
| *Pseudomonas taiwanensis* |  |  |  |  |  |  |  | + |  |  |  | Chen et al., 2016 |
| *Pseudomonas protegens* |  |  |  |  |  |  |  | + |  |  |  | Vacheron et al., 2019 |
| *P. brassicacearum* |  |  | + | + | + | + | + | + |  |  |  | Gislason and Kievit 2019 |
| ***Azoarcus*** |  |  |  |  |  |  |  |  |  |  |  |  |
| *Azoarcus oleaius* |  |  |  |  |  |  |  | + |  |  |  | Jiang et al. 2019 |
| *Azoarcus* sp. BH72. |  |  |  |  |  |  |  | + |  |  |  | Shidore 2012 |
| *Azoarcus* sp. CIB |  |  | + | + |  | + |  | + |  |  |  | Martín-Moldes et al. 2015 |
| ***Rhizobium*** |  |  |  |  |  |  |  |  |  |  |  |  |
| *Rhizibiaceae family* (fourteen Rhizobiales genomes) | + | + | + | - | + | + | - | + | - | - | - | Black et al. 2012 |
| *Rhizobium leguminosarum* bv. Trifolii | + |  | + |  |  |  |  |  |  |  |  | Mazur et al., 1998 |
| *R. leguminosarum* bv. Viciae (strain A34, 3841), UPM791, bv. Trifolii strain TA1 |  | + | + |  |  |  | +  (3841) |  |  |  |  | Finnie et al.1997; |
| *Rhizobium tropici* PRF 81 |  |  | + | + | + | + |  |  |  |  |  | Stanfield et al. 1988; Pinto et al. 2009 |
| *Rhizobium etli* |  |  | + |  |  | + |  |  |  |  |  | Pérez-Mendoza et al., 2005 |
| *Rhizobium* sp. NGR234 |  |  |  |  | + |  |  |  |  |  |  | Bartsev et al. 2004 |
| *R. leguminosarum* Norway |  |  | + |  |  | + | + | + |  |  |  | Liang et al. 2018 |
| ***Sinorhizobium*** |  |  |  |  |  |  |  |  |  |  |  |  |
| *Sinorhizobium meliloti, Sinorhizobium* NGR 234 |  |  | + | + | + | + |  |  |  |  |  | Stanfield et al. 1988; Pinto et al. 2009 |
| *Sinorhizobium* HH103, USDA257 and NGR234 |  |  |  |  | + |  |  |  |  |  |  | López-Baena et al. 2016 |
| *Ensifer fredii* |  |  | + | + | + | + |  |  |  |  |  | Dang et al. 2019 |
| ***Bradyrhizobium*** |  |  |  |  |  |  |  |  |  |  |  |  |
| *Bradyrhizobium* |  |  | + | + |  |  |  |  |  |  |  | Black et al. 2012 |
| *Bradyrhizobium japonicum* |  |  |  |  | + |  |  |  |  |  |  | Nelson and Sadowsky 2015 |
| *Bradyrhizobial* strain USDA110 *Bradyrhizobium* sp. BTAi1 |  |  |  |  | + | + |  |  |  |  |  | Nelson and Sadowsky 2015 |
| Bradyrhizobium elkanii |  |  |  |  | + |  |  |  |  |  |  | Okazaki et al., 2009; Sanchez et al., 2009 |
| Bradyrhizobium sp. DOA9 |  |  |  |  | + |  |  |  |  |  |  | Songwattana et al. 2017 |
| *Bradyrhizobium* sp. SUTN9-2 |  |  |  |  | + | + |  | + |  |  |  | Piromyou et al., 2015 |
| ***Mesorhizobium*** |  |  |  |  |  |  |  |  |  |  |  |  |
| *Mesorhizobium loti* MAFF303099 |  | + |  | + |  |  |  |  |  |  |  | Black et al. 2012 |
| *Mesorhizobium loti* strain R7A |  |  |  |  |  | + |  |  |  |  |  | Hubber et al., 2004 |
| ***Other Rhizobia*** |  |  |  |  |  |  |  |  |  |  |  |  |
| *Neorhizobium galegae* |  |  | + |  |  | + |  | + |  |  |  | Osterman 2015 |
| *Dugesia japonica* MF79 |  |  |  | + | + |  |  |  |  |  |  | Teixeira et al. 2021 |
| *Ensifer meliloti* 1021 |  |  |  |  |  | + |  |  |  |  |  | Carvalho et al. 2010 |
| ***Herbaspirillum*** |  |  |  |  |  |  |  |  |  |  |  |  |
| *Herbaspirillum seropedicae* strain SmR1, *H. rubrisubalbicans* M1, HsOs34, HsOs45, HCF444, and HYR522, |  |  |  |  | + |  |  |  |  |  |  | Piromyou et al. 2015; Monteiro et al., 2012a |
| *Herbaspirillum* strains *AzospB510*, *Kp342*, and *GdPAI5* |  |  |  |  | + |  |  |  |  |  |  | Straub et al. 2013 |
| *Herbaspirillum* strain HfGSF30 |  | + |  |  |  |  |  | + |  |  |  | Straub et al. 2013 |
| ***Others*** |  |  |  |  |  |  |  |  |  |  |  |  |
| *Azospirillum brasilense* Az39 |  |  |  |  |  |  |  | + |  |  |  | *(reference )*. |
| *Kosakonia radicincitans* |  |  | + | + | + | + |  | + |  |  |  | Becker et al. 2018 |
| Paraburkholderia kururiensis type strain KP23^T^ |  |  |  |  | + | + | + | + |  |  |  | Levy et al. 2018 |
| Paraburkholderia *kururiensis* M130 and ATSB13T |  |  |  |  | + |  | + |  |  |  |  | Dias et al. 2018 |
| *Xanthomonas* |  |  | + | + |  |  |  | + |  |  |  | Li et al. 2020 |
| *Klebsiella* D5A | + | + | + | + | + |  | + | + |  |  |  | Liu et al. 2016 |
| *P. ananatis* |  |  |  |  |  |  |  | + |  |  |  | Sheibani-Tezerji et al. 2015 |
| Flavobacterium |  |  |  |  |  |  |  |  |  |  | + | Nakane et al. 2013 |
| *Enterobacter* sp. SA187 | + | + |  | + |  |  |  | + |  |  |  | Andres-Barrao C. et al*.* 2017 |
| *Hartmannibacter diazotrophicus* strain E19T | + | + | + | + | + | + |  | + |  |  |  | Suarez 2019 |
| *Nitrospirillum amazonense* variety RB867515 | + | + |  |  |  |  |  |  |  |  |  | Terra et al. 2019 |
| *B. phytofirmans* strains |  |  | + | + | + | + |  | + |  |  |  | Mitter et al. 2013 |
| *B. phytofirmans* PsJN |  |  |  | + |  | + |  |  |  |  |  | Sheibani-Tezerji et al. 2015 |
| *Stenotrophomonas rhizophila* DSM14405T |  |  |  | + |  | + | + | + |  |  |  | Alavi et al. 2014 |
