# Supplementary material for: Secretory molecules from secretion systems fine-tune the host-beneficial bacteria (PGPRs) interaction
Source: Front Microbiol. 2024 Feb 26;15:1355750. doi: 10.3389/fmicb.2024.1355750 (PMC10925705; doi:10.3389/fmicb.2024.1355750)
Supplement: Supplementary file 2 [file Table_2.doc]

**Supplementary Table 2**

General Secretion systems (SEC) in PGPRs

| **S. No.** | **PGPR** | **Type of Plant associated Bacteria** | **Type of secretion system** | **Function of secretion system/**  **secreted Effectors** | **Host** | **Some product** | **References** |
| --- | --- | --- | --- | --- | --- | --- | --- |
| 1. | *Rhizobium leguminosarum* bv. Trifolii | Symbiotic | Sec system | Colonization | Legumes | Cellulase | Robledo et al., 2008 |
| 2. | *Bacillus amyloliquefaciens* FZB42 | Rhizospheric | Sec , TAT pathway, T1SS,T2SS | Secrete various proteins | Beet | - | Kierul et al. 2015 |
| 3. | *P. fluorescens* Pf0-1 | Rhizospheric | outer membrane export systems, (MTB (Main terminal branch), FUP (Fimbrial Usher porin), AT (Autotransporter) and TPS (Two partner secretion families)), in several copies (2-9), three or four ABC-type protein secretory systems, flagellar secretion system, Sec system | - | Loam soil | - | Ma Q et al., 2003 |
| 4. | *Nitrospirillum amazonense* variety RB867515 | Endophytic Diazotrophic | Sec and TAT | - | Sugarcane | - | Terra et al. 2019 |
