# Supplementary material for: Secretory molecules from secretion systems fine-tune the host-beneficial bacteria (PGPRs) interaction
Source: Front Microbiol. 2024 Feb 26;15:1355750. doi: 10.3389/fmicb.2024.1355750 (PMC10925705; doi:10.3389/fmicb.2024.1355750)
Supplement: Supplementary file 3 [file Table_3.doc]

**Supplementary Table 3**

TAT secretion systems in PGPRs.

| **S. No.** | **PGPR** | **Type of Plant associated Bateria** | **Type of Secretion system** | **Function of Secretion system/**  **secreted Effectors** | **Host** | **Some product** | **References** |
| --- | --- | --- | --- | --- | --- | --- | --- |
| 1. | *R. leguminosarum* and Rhizobiaceae family | Symbiotic | TAT | Role in Nitrogen fixation, aerobic and anaerobic growth of bacteria, symbiosis and root nodule development, cell component synthesis. | Legumes | - | Jack et al. 2011; Pickering et al. 2012 |
| 2. | *Mesorhizobium loti* MAFF303099 | Symbiotic | TAT | Induce nodulation initiation | Legumes | - | Black et al 2012 |
| 3. | *R. leguminosarum bv. Viciae* UPM791, 3841 | Symbiotic | TAT | Nitrogen fixation, Infection and colonization, secrete symbiosis specific proteins | Legumes (Pea) | - | Meloni et al., 2003; Krehenbrink & Downie, 2008 |
| 4. | *Bacillus megaterium* STB1 | Rhizosphere | Sec system, TAT, T7SS | - | Soil | - | Nascimento et al., 2020 |
| 5. | *Bacillus amyloliquefaciens* FZB42 | Rhizosphere | SEC, TAT pathway, T1SS,T2SS | Secrete various proteins | Beet | PhoD), YfkN, Qcr | Kierul et al. 2015 |
| 6. | *Bacillus velezensis* 9D-6 | Rhizosphere | Type VII/ESX, TAT system, and SecYEG translocon | Not known | Potato | - | Grady et al. 2019 |
| 7. | *B. amyloliquefaciens* subsp. plantarum UCMB5113 | Rhizosphere | Sec and TAT system | - | Soil | - | Niazi et al. 2014 |
| 8. | *Pseudomonas* sp. UW4 | Rhizosphere | Sec, Tat, Type I, II, III, V and VI, MscL protein channel | - | *Phragmites australis*  (Common Reeds) | - | Duan et al. 2013 |
| 9. | *P. fluorescens* Pf0-1 | Rhizosphere | TAT, holin-like proteins, MscL, ABC protein transporters, fimbrial Usher proteins (FUP) | Fimbriae biogenesis and sometimes in biofilm formation (FUP) | Loam soil | -. | Ma Q et al. 2003 |
| 10. | *Klebsiella* D5A | Rhizospheric soil | Types I, II, III, V and VI, Tat (twin arginine translocation), and Sec (general secretory pathway) | - | *Testuca arundinacea* L. | - | Liu et al. 2016 |
| 11. | *Herbaspirillum frisingense* GSF30T | Endophytes | type I, type VI, Sec-SRP and the Tat | - | Miscanthus sacchariflorus | - | Straub et al. 2013 |

It is involved in carrying protein across the plasma membrane and in other processes like nitrogen-fixation, symbiosis, bacterial pathogenesis, ATP utilization, cell wall formation, biofilm development, heavy metal resistance etc (Patel et al., 2014).
