# Supplementary material for: Secretory molecules from secretion systems fine-tune the host-beneficial bacteria (PGPRs) interaction
Source: Front Microbiol. 2024 Feb 26;15:1355750. doi: 10.3389/fmicb.2024.1355750 (PMC10925705; doi:10.3389/fmicb.2024.1355750)
Supplement: Supplementary file 4 [file Table_4.doc]

**Supplementary Table 4**

Type 1 (T1SS) secretion system in PGPRs.

| **S. No.** | **PGPR** | **Type of Plant associated Bacteria** | **Type of Secretion system** | **Function of Secretion system/**  **secreted Effectors** | **Host** | **Some product** | **References** |
| --- | --- | --- | --- | --- | --- | --- | --- |
| 1. | *Rhizibiaceae family* | Symbiont | GEP, TAT, Type I (prsDE encoded secretion system) Type III, Type IV and Type VI | Symbiosis, attachment, infection proteins, Nitrogen fixation | Legumes | NodO, Glycanases | Finnie et al.1997; Mazur et al., 1998 |
| 2. | *R. leguminosarum* bv. viciae strain 3841, A34, bv. Trifolii strain TA1 | Symbiont | PrsD-PrsE type I secretion system | Nitrogen fixation (Not in 3841), metalloprotease, glycosyl hydrolase, cadherins and a nucleoside diphosphate kinase, infection threads development or senescing plant cells, bacteriocin/antibiotic production | Legumes | NodO, PlyA, PlyB, PlyC, RapA2, RapB and RapC,RL2412, RL2961, pRL90140 and pRL100307 | Krehenbrink and Downie 2008 |
| 3. | *R. leguminosarum* Norway | Symbiont | Type I, IV, V.VI | Biofilm formation | Lotus | RTX toxins | Liang et al. 2018 |
| 4. | *Rhizobium tropici* PRF 81 | Symbiont | Type I, II, III, IV | resistance towards macrolide antibiotic tylosin, biofilm formation | Legumes | macrolide antibiotic tylosin, (1-2)-β-glucanase | Stanfield et al. 1988; Pinto et al. 2009 |
| 5. | *S. meliloti* | Symbiont | 1. Type I; 2. T1SS and ExpD1D2 T1SS | 1. biofilm formation; 2. No role in symbiosis | *Medicago sativa* | 1.NdvA protein;  2. ExoK and ExpE1 respectively | 1. Stanfield et al. 1988; Pinto et al. 2009; 2. Moreira et al., 2000 |
| 6. | *Sinorhizobium meliloti, Rhizobium leguminosarum, Bradyrhizobium* | Symbiont | PrsD-PrsE type I secretion system | 1.colonization, attachment and biofilm formation  2. attachment and biofilm formation  3. attachment to root hairs  4. calcium binding proteins | Legumes | 1.glycanases 2. adhesins (like Rap) 3.Rhicadhesin  4. cadherins | Russo et al., 2006; Krehenbrink & Downie, 2008; Mongiardini et al., 2008 ; Smit et al., 1992 ;Dardanelli et al., 2003 |
| 7. | *Pseudomonas* model strains WCS417 | Rhizospheric | two complete T1SS loci | - | Wheat | 1. lipase and alkaline protease AprA,  2. hemophore HasA | Berendsen 2015 |
| 8. | *Pseudomonas* (WCS374 and WCS358) | Rhizospheric | two complete T1SS loci | - | Potato | alkaline protease aprA and a lipase by first gene cluster. No information about second gene cluster of WCS374; of both clusters of WCS358 | Berendsen 2015 |
| 9. | *Pseudomonas* sp. UW4 | Rhizospheric | Sec, Tat, Type I, II, III, V and VI, MscL protein channel | - | *Phragmites australis*  (Common Reeds) | - | Duan et al. 2013 |
| 10. | P. putida KT2440 | Soil isolate,Rhizospheric | type I LapBC secretion system | - | crop plants, such as corn plants, wheat, strawberry, sugarcane and spinach | LapA protein synthesizing in biofilm | [Hinsa et al*.* 2003](javascript:;) |
| 11. | P. putida W619 | Endophyte | type I secretion system | - | Populus trichocarpa x deltoides cv. "Hoogvorst" | putative adhesin (PputW619_3808) and a surface-adhesion calcium-binding outer membrane protein | Wu et al. 2010 |
| 12. | *B. phytofirmans (in different strains)* | PGPR/Rhizospheric | T1SS, T2SS, T3SS, T4SS, and T6SS | - | - | - | Mitter et al. 2013 |
