# Supplementary material for: Secretory molecules from secretion systems fine-tune the host-beneficial bacteria (PGPRs) interaction
Source: Front Microbiol. 2024 Feb 26;15:1355750. doi: 10.3389/fmicb.2024.1355750 (PMC10925705; doi:10.3389/fmicb.2024.1355750)
Supplement: Supplementary file 5 [file Table_5.doc]

**Supplementary Table 5**

Type 2 secretion systems in PGPRs.

| **S. No.** | **PGPR** | **Type of Plant associated Bacteria** | **Type of Secretion system** | **Function of Secretion system/**  **secreted Effectors** | **Host** | **Some product** | **References** |
| --- | --- | --- | --- | --- | --- | --- | --- |
| 1. | *Bradyrhizobium*,  *M. loti* and *Sinorhizobium*  NGR 234 | Symbiont | T2SS | - | Legumes | - | [33] |
| 2. | *S. rhizophila* DSM14405T | Symbiont | T2SS, 4,5,6 | - | Legumes | - | [62] |
| 3. | *D. japonica* MF79 | Symbiont | Type 2, 3 | infection by suppressing the flg22 response | Legumes | - | [63] |
| 4. | *Pseudomonas* WCS417 | Rhizospheric | Two T2SS loci;  One  T2SS loci | - | Wheat | - | [53] |
| 5. | *Pseudomonas* WCS374 and WCS358 strain | Rhizospheric | One  T2SS loci | - | Potato | PhoX-type phosphatase UxpB under phosphate-limited conditions in WCS358 | [53] |
| 6. | *Pseudomonas* sp. UW4 | Rhizospheric | T2SS | - | *Phragmites australis*  (Common Reeds) | - | [54] |
| 7. | *Enterobacter* sp. SA187 | Endophyte | Sec, TAT, T2SS, T6SS | - | *Indigofera argentea* | - | [22] |
| 8. | *B. phytofirmans* PsJN | Endophyte | type II and type IV | - | Potato | - | [64] |
| 9. | *Xanthomonas* | Endophytic | T1SS, T2SS, T6SS | - | *Lolium perenne* L. cv. Alto | - | [94] |
