# Supplementary material for: Secretory molecules from secretion systems fine-tune the host-beneficial bacteria (PGPRs) interaction
Source: Front Microbiol. 2024 Feb 26;15:1355750. doi: 10.3389/fmicb.2024.1355750 (PMC10925705; doi:10.3389/fmicb.2024.1355750)
Supplement: Supplementary file 6 [file Table_6.doc]

**Supplementary Table 6**

Type 3 secretion systems in PGPRs.

| **S.No.** | **PGPR** | **Type of Plant associated Bacteria** | **Type of Secretion system** | **Function of Secretion system/**  **secreted Effectors** | **Host** | **Some product** | **References** |
| --- | --- | --- | --- | --- | --- | --- | --- |
| 1. | *Rhizobia, Bradyrhizobium japonicum* Blr1693 Bl18244 | Symbiont | T3SS | Symbiotic association | Legumes | NopD | Nelson and Sadowsky, 2015 |
| 2. | *B. japonicum* USDA110, *Mesorhizobium loti* MAFF303099, *Rhizobium etli* CNPAF512, *Rhizobium* sp. NGR234 and *S. fredii* strains HH103 and USDA257 | Symbiont | T3SS | symbiotic function, enhancing defense responses | Legumes | - | Deakin and Broughton, 2009 |
| 3. | *Rhizobium tropici* PRF 81 | Symbiont | T3SS | flagellum-related genes | Legumes | - | Pinto et al. 2009 |
| 4. | *Rhizobium* sp. NGR234 | Symbiont | T3SS | PR genes suppression | Legumes | - | Bartsev et al. 2004 |
| 5. | *Sinorhizobium* (*Ensifer) fredii* HH103 | Symbiont | T3SS | Formation of infection thread, Nodule formation | Legumes | NopL, NopM and NopP | Jiménez-Guerrero et al. 2015 |
| 6. | *Sinorhizobia* NGR234, *S. fredii* USDA257 | Symbiont | T3SS | Nodulation | Legumes | Sinorhizobial proteins | López-Baena et al. 2016 |
| 7. | Bradyrhizobium elkanii | Symbiont | T3SS | Nodulation, symbiosis, intracellular infection | Legumes | NopE1 and NopE2, GunA2 and probably NopL, NopM and NopT homologues | Okazaki 2013 |
| 8. | Bradyrhizobium sp. DOA9 | Symbiont | T3SS | Symbiotic interaction | Legumes | - | Songwattana et al. 2017 |
| 9. | *Bradyrhizobium* sp. SUTN9-2 | Symbiont | T3SS (rhcJ) and T4SS (virD4) | infection of rice and leguminous crop, over  coming plant defense | *Aeschynomene americana* L. | - | Piromyou et al. 2015 |
| 10. | *P. fluorescens* KD | Rhizospheric | T3SS | biocontrol activity | Soil | - | Rezzonico et al. 2004 |
| 11. | 1*. Pseudomonas chlororaphis* GP72,  2. *Pseudomonas fluorescens* Pf-5,  3. *Pseudomonas aeruginosa* M-18,  4. *Pseudomonas stutzeri* A1501 | 1,2,3. Rhizospheric  4. Endophytic | Type I, type II, Type III (Only M-18), type IV, type V, and type VI, chaperone-usher secretion system and TAT system | - | 1. Green Pepper  2. Cotton  3. Sweet melon  4. Rice | - | Shen et al. 2013 |
| 12. | 1. *P. fluorescens* strains like WH6, 2. Q8r1-96, and 3. BBc6R8 | 1., 2. Rhizosphere  3.  ectomycorrhizal fungus Laccaria bicolor | T3SS | Biocontrol, Myccorhization (BBc6R8) | 1., 2. Wheat  3. Laccaria bicolor | 2. RopM, RopAA, and RopB | Cusano et al. 2011 |
| 13. | 1. *Pseudomonas* strains WCS417 and,  2. WCS374 | Rhizospheric | T1SS,T2SS,T3SS,T5SS,T6SS | - | 1. Wheat  2. Potato | Secrete ExoU and HopJ | DebRoy et al. 2004 |
| 14. | *P. fluorescens* C7R12 | Rhizospheric | T3SS | Mycorrhization | Flax | - | Viollet et al. 2017 |
| 15. | *P.*  *fluorescens* SBW25 | Phylloplanic | T3SS | - | Sugarbeet | - | Rezzonico et al. 2004 |
| 16. | *Pseudomonas fluorescens* 2P24 | Soil | T3SS | Effectors secretion | Wheat | - | Liu et al. 2016 |
| 17. | *Pseudomonas* UW4 | Rhizospheric | Type I, II, III, and IV secretion system | - | *Phragmites australis*  (Common Reeds) | - | Duan et al. 2013 |
| 18. | *Pseudomonas syringa*e strain 260-02 | PGPR | I, III, IV, V, VI and VII | not secrete toxins, having more biocontrol activity | Malus pumila (Apple) | - | Passera et al. 2019 |
| 19. | *P. brassicacearum* | PGPR/Rhizospheric | T1SS,T2SS,T3SS,T5SS,T6SS | - | Different sources | HopAA | Gislason and Kievit 2019. |
| 20. | *H. rubrisubalbicans* HsSmR1, M1, HsOs34, HsOs45, HCF444, HrM1 and HYR522 | Endophytic | Secretion systems (I, II, III, IV, and VI), particularly of type III (T3SS) and VI (T6SS) | Endophytic colonization, Host immune suppression | Grasses | - | Monteiro et al., 2012a |
| 21. | *Ensifer fredii* | Endophytic | type III, IV, V, and VI | host-specific nodulation, exporting effector proteins and host defense reactions | Soyabean nodules | - | Dang et al. 2019 |
| 22. | *Kosakonia radicincitans* DSM 16656T | Rhizospheric | I, II, III, IV, and VI | Motility | Wheat | - | Becker et al. 2018 |
| 23. | Paraburkholderia kururiensis type strain KP23^T^, *P. kururiensis* M130 | Polluted soil | III, IV, V, VI | colonization and bacterial adaptation | - | - | Levy et al. 2018 |
| 24. | Pantoea agglomerans strain PG734, P5, IG1 and 190 | Rhizospheric | Type III | - | Soil | - | Shariati J et al. 2017 |
| 25. | *Burkholderia phytofirmans* PsJN | Endophyte | Type I, type II, type III, type IV, and type VI | Endophytic colonization | Onion | - | Piromyou et al. 2015 |
