# Supplementary material for: Secretory molecules from secretion systems fine-tune the host-beneficial bacteria (PGPRs) interaction
Source: Front Microbiol. 2024 Feb 26;15:1355750. doi: 10.3389/fmicb.2024.1355750 (PMC10925705; doi:10.3389/fmicb.2024.1355750)
Supplement: Supplementary file 7 [file Table_7.doc]

**Supplementary Table 7.**

T4SS Secretion systems in PGPRs

| **S.No.** | **PGPR** | **Type of Plant associated Bacteria** | **Type of Secretion system** | **Function of Secretion system/**  **secreted Effectors** | **Host** | **Some product** | **References** |
| --- | --- | --- | --- | --- | --- | --- | --- |
| 1. | *Rhizobium tropici* PRF 81; *R. etli* and *S. meliloti* | Symbiont | T1SS, T2SS,T3SS T4SS | conjugation-transfer of symbiotic plasmids | Legumes | - | Pinto et al. 2009 |
|  | *R. leguminosarum* Norway | Symbiont | Type I, IV, V.VI | - | Lotus | - | Liang et al. 2018 |
| 2. | *Mesorhizobium loti* strain R7A | Symbiont | T4SS | Symbiotic infection 1. ubiquitinylation of plant proteins for degradation via proteasomes,  2. Protease, | Legumes | 1. Msi061 2. Msi059 | Hubber et al., 2004 |
| 3. | 1. *E. meliloti* 1021  2. *S. meliloti* | Symbiont | 1. T4SS 2. T1SS, T2SS,T3SS T4SS | conjugation but not for symbiosis | Legumes | - | Carvalho et al. 2010 |
| 4. | *Bradyrhizobium* sp. SUTN9-2 | Symbiont | T4SS | - | Legumes | - | Piromyou et al., 2015 |
| 5. | *Bradyrhizobial* strain USDA110 *Bradyrhizobium* sp. BTAi1 | Symbiont | T4SS | - | Legumes | - | Nelson and Sadowsky 2015 |
| 6. | *Pseudomonas viridiflava* CDRTc14 | Endophyte | T1SS, T2SS,T4SS, T5SS, T6SS, and T7SS | - | *Lepidium draba* or grapevine | - | Samad et al., 2017 |
| 7. | *Herbaspirillum* strains. *AzospB510*, *Kp342*, and *GdPAI5* | Endophyte | T4SS | - | Grasses | - | Straub et al. 2013 |
| 8. | Paraburkholderia kururiensis type strain KP23^T^ | Polluted soil | T3SS,T4SS, T5SS, T6SS | microbial competition | - | - | Dias et al. 2018 |
| 9. | *B. phytofirmans* PsJN | Root endophyte | T2SS, T4SS | - | Onion | - | Sheibani-Tezerji et al. 2015 |
