# Supplementary material for: Secretory molecules from secretion systems fine-tune the host-beneficial bacteria (PGPRs) interaction
Source: Front Microbiol. 2024 Feb 26;15:1355750. doi: 10.3389/fmicb.2024.1355750 (PMC10925705; doi:10.3389/fmicb.2024.1355750)
Supplement: Supplementary file 8 [file Table_8.doc]

**Supplementary Table 8.**

Type 5 Secretion system in PGPRs.

| **S.No.** | **PGPR** | **Type of Plant associated Bacteria** | **Type of Secretion system** | **Function of Secretion system/**  **secreted Effectors** | **Host** | **Some product** | **References** |
| --- | --- | --- | --- | --- | --- | --- | --- |
| 1. | *R. leguminosarum* bv. Viciae strain 3841 | Symbiont | TAT,T1SS, T5SS | no role in symbiotic interaction | Legumes | - | Krehenbrink & Downie, 2008 |
|  | *R. leguminosarum* Norway | Symbiont | Type I, IV, V.VI | - | Lotus | - | Liang et al. 2018 |
| 2. | *Pseudomonas* 1.WCS417, 2.WCS358,  3. WCS374, | Rhizospheric | T1SS,T2SS,T3SS,T5SS,T6SS | colonization | 1. Wheat  2., 3. Potato | TpsA | Berendsen 2015 |
| 3. | *Pseudomonas* UW4 | Rhizospheric | Sec, TAT, Type I, II, III, IV, V secretion system | twitching, swarming, and swimming motilities | *Phragmites australis*  (Common Reeds) | - | Duan et al. 2013 |
| 4. | *P. putida* W619 | Endophyte | T1SS,T5SS,T6SS | putative adhesin, a surface-adhesion calcium-binding outer membrane-like protein | Populus trichocarpa×deltoides cv. ‘Hoogvorst,’ | ndvB secreting b-(1,2)-glucan | Wu et al. 2010 |
| 5. | 1. *P. kururiensis* KP23T,  2., 3. M130 and ATSB13T | Polluted soil | 1. T3SS,T4SS, T5SS,T6SS   2.,3. T3SS, T5SS | - | - | - | Dias et al. 2018 |
