# Supplementary material for: Secretory molecules from secretion systems fine-tune the host-beneficial bacteria (PGPRs) interaction
Source: Front Microbiol. 2024 Feb 26;15:1355750. doi: 10.3389/fmicb.2024.1355750 (PMC10925705; doi:10.3389/fmicb.2024.1355750)
Supplement: Supplementary file 9 [file Table_9.doc]

**Supplementary Table 9.**

T6SS secretion systems in PGPRs.

| **S.No.** | **PGPR** | **Type of Plant associated Bacteria** | **Type of Secretion system** | **Function of Secretion system/**  **secreted Effectors** | **Host** | **Some product** | **References** |
| --- | --- | --- | --- | --- | --- | --- | --- |
| 1. | *Rhizobium* | Symbiont | T6SS | Nitrogen fixation | Legumes | RbsB | Shyntum et al. 2014 |
| 2. | *R. leguminosarum* Norway | Symbiont | Type I, IV, V.VI | - | Lotus | - | Liang et al. 2018 |
| 3. | *Pseudomonas fluorescens* MFE01 | Environment | T6SS | 1. antibacterial activity 2. bacterial motility | 1. Hcp2 or Hcp3 (antibacterial activity) 2. Hcp1 (bacterial motility) | - | Decoin et al. 2014 |
| 4. | *Pseudomonas protegens* | Soil | T6SS | - | - | - | Vacheron et al., 2019 |
| 5. | *P. fluorescens* Pf29Arp | Soil | T6SS | Adaptation, Biocontrol | - | - | Marchi et al., 2013 |
| 6. | *P. taiwanensis* | Soil | T6SS | Biocontrol | - | pyoverdin | Chen et al., 2016 |
| 7. | *Pseudomonas* W619 | Endophyte | T1SS,T5SS,T6SS | - | Populus trichocarpa×deltoides cv. ‘Hoogvorst,’ | - | Wu et al. 2010 |
| 8. | *P. putida* KT2440 | Soil isolate,  Rhizospheric | T1SS,T2SS,T6SS | Biocontrol | crop plants, such as corn | - | Bernal et al., 2017 |
| 9. | *Pseudomonas* 1.WCS417, 2.WCS358,  3. WCS374, | Rhizospheric | T1SS,T2SS,T3SS,T5SS,T6SS |  | 1. Wheat  2., 3. Potato |  | Bendersen et al. 2015 |
| 10. | *Pseudomonas* UW4 | Rhizospheric | Sec, TAT, Type I, II, III, IV, V secretion system | Biocontrol, colonization | *Phragmites australis*  (Common Reeds) | RhsA and Ghh1 | Vacheron et al., 2019 |
| 11. | *Azoarcus oleaius* | Soil | T6SS | - | - | - | Jiang et al. 2019 |
| 12. | *Azoarcus* sp. BH72. | Endophytic | T6SS | limit endophytic colonization | Kallar grass | - | Shidore 2012 |
| 13. | *Azoarcus* sp. CIB | Free living/endophyte | TISS, T2SS, T4SS and T6SS systems | - | - | - | Martín-Moldes et al. 2015 |
| 14. | *Enterobacter* sp. SA187 | Endophyte | Sec, TAT, T2SS, T6SS | - | *Indigofera argentea* | - | Andres-Barrao C. et al*.* 2017 |
| 15. | *Pseudomonas* UW4 | Rhizospheric | Sec, TAT, Type I, II, III, IV, V secretion system | - | *Phragmites australis*  (Common Reeds) | - | Duan et al. 2013 |
| 16. | *Herbaspirillum* strain HfGSF30 | Endophyte | T6SS | - | Grass | - | Straub et al. 2013 |
| 17. | *P. kururiensis* KP23T, M130, ATSB13T | Polluted soil | T3SS,T4SS, T5SS,T6SS | - | - | hcp and vgrG | Dias et al. 2018 |
| 18. | *Neorhizobium galegae* | Endophyte | TISS, T4SS, T6SS system | Not involved in symbiosis | *Galega orientalis* Lam. and *G. officinalis* L. | - | Osterman 2015 |
| 19. | *Azospirillum brasilense* Az39 | Endophyte | T6SS | lipid production, carbohydrates, and photosynthetic pigments, attachment, IAA production, bio-control | Wheat | - | Rivera et al. 2014 |
| 20. | *P. ananatis* | Rhizospheric | T6SS | competition, fitness or niche adaptation | Green onion | - | Sheibani-Tezerji et al. 2015 |
| 21. | *Burkholderia phytofirmans* PsJN, *Burkholderia* spp. strain JK006, *Azospirillum lipoferum* 4B, *Enterobacter cloacae* ENHKU01, *Klebsiella pneumoniae* 342, *Pseudomonas putida* W619*, Enterobacter spp.* 638, *Azoarcus* spp. BH72, and *Serratia proteamaculans* 568 | Endophytic | T6SS, RND system MFP subunit | - | - | - | Ali et al. 2014 |
